# Rapid retreat of Thwaites Glacier in the pre-satellite era

**DOI:** 10.1038/s41561-022-01019-9

**Published:** 2022-09-05

**Authors:** Alastair G. C. Graham, Anna Wåhlin, Kelly A. Hogan, Frank O. Nitsche, Karen J. Heywood, Rebecca L. Totten, James A. Smith, Claus-Dieter Hillenbrand, Lauren M. Simkins, John B. Anderson, Julia S. Wellner, Robert D. Larter

**Affiliations:** 1 College of Marine Science, University of South Florida, St Petersburg, FL USA; 2 Department of Marine Sciences, University of Gothenburg, Gothenburg, Sweden; 3 British Antarctic Survey, Natural Environment Research Council, Cambridge, UK; 4 Lamont-Doherty Earth Observatory, Columbia University, Palisades, NY USA; 5 School of Environmental Sciences, University of East Anglia, Norwich, UK; 6 Department of Geological Sciences, University of Alabama, Tuscaloosa, AL USA; 7 Department of Environmental Sciences, University of Virginia, Charlottesville, VA USA; 8 Department of Earth, Environmental and Planetary Sciences, Rice University, Houston, TX USA; 9 Department of Earth and Atmospheric Sciences, University of Houston, Houston, TX USA

**Keywords:** Cryospheric science, Geomorphology, Climate change, Ocean sciences

## Abstract

Understanding the recent history of Thwaites Glacier, and the processes controlling its ongoing retreat, is key to projecting Antarctic contributions to future sea-level rise. Of particular concern is how the glacier grounding zone might evolve over coming decades where it is stabilized by sea-floor bathymetric highs. Here we use geophysical data from an autonomous underwater vehicle deployed at the Thwaites Glacier ice front, to document the ocean-floor imprint of past retreat from a sea-bed promontory. We show patterns of back-stepping sedimentary ridges formed daily by a mechanism of tidal lifting and settling at the grounding line at a time when Thwaites Glacier was more advanced than it is today. Over a duration of 5.5 months, Thwaites grounding zone retreated at a rate of >2.1 km per year—twice the rate observed by satellite at the fastest retreating part of the grounding zone between 2011 and 2019. Our results suggest that sustained pulses of rapid retreat have occurred at Thwaites Glacier in the past two centuries. Similar rapid retreat pulses are likely to occur in the near future when the grounding zone migrates back off stabilizing high points on the sea floor.

## Abstract (web summary)

The Thwaites Glacier grounding zone has experienced sustained pulses of rapid retreat over the past two centuries, according to sea floor observations obtained by an autonomous underwater vehicle.

## Main

**Fig. 1:**
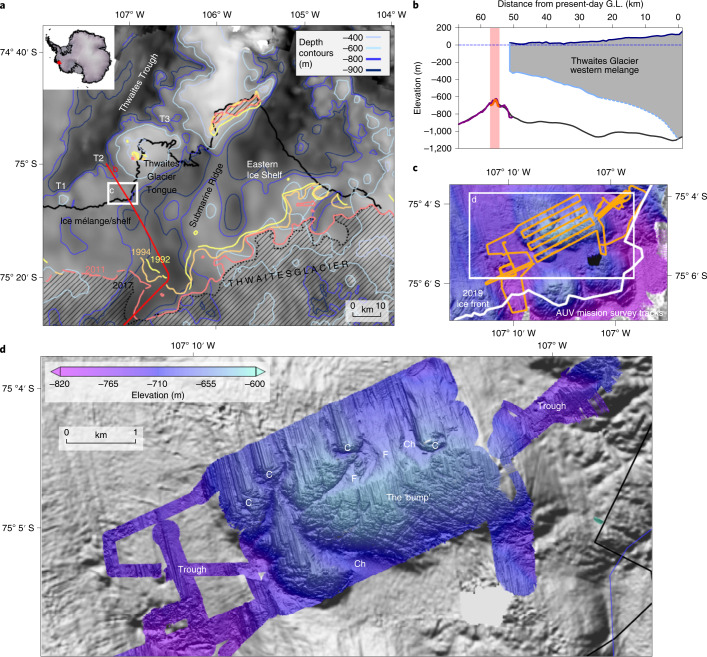
Location map and context of the Thwaites Glacier study site. **a**, Map of the southern Amundsen Sea embayment in the vicinity of Thwaites Glacier. Bathymetry/topography covering marine, sub-ice shelf and grounded glacier from ref. ^37^. Previous grounding-line positions, as well as pinning points at the termini of the TGT and Eastern Ice Shelf, are shown in colour. Modern region of grounding hatched. T1-T3: tributary troughs. **b**, Long profile showing study site context. Pink bar and orange-shaded bathymetry show area of new survey. G.L., grounding line. **c**, AUV survey tracks at the apex of the Thwaites western ice mélange and TGT. **d**, AUV multibeam swath bathymetry of 1.5 m resolution superimposed on a hillshade-rendered image of shipborne EM122 swath bathymetry collected on the same research cruise. F, fan; C, crag; Ch, channel.

Ice loss from West Antarctica’s second largest marine ice stream, Thwaites Glacier, is currently a major uncertainty for future sea-level projections^1–3^. Its bed deepens upstream to >2 km below sea level^4^, and warm, dense, deep water delivers heat to the present-day ice-shelf cavity, melting its ice shelves from below^5^. Together, these conditions make Thwaites Glacier susceptible to runaway retreat^6^. Satellite radar observations of change in the ice stream show that its fast-flowing trunk has sped up, thinned and widened since 2011, while there has been spatially variable grounding-line retreat^7,8^. Such changes have occurred as a consequence of reduced buttressing from weakened contact with a shallow ridge at the Eastern Ice Shelf terminus^8–10^, and fragmentation and subsequent detachment of the Thwaites Glacier Tongue (TGT) from a sea-bed pinning point^11^ (Fig. 1a). As the stabilizing effects of these ice-shelf pinning points lessen^12^, future retreat of Thwaites becomes increasingly predicated on processes that occur in the grounding zone—the region in which the glacier comes afloat in the ocean. Sea-floor topography can help stabilize ice sheets against grounding-line retreat^13^, and grounding-line migration affects ice-sheet stability on timescales spanning months to millennia^14^. Currently, Thwaites is grounded on prominent sea floor ridges in a number of places^15^, and the evolution of grounding-zone processes here is critical to future ice retreat^16,17^. However, the processes operating at marine ice-stream margins are poorly resolved, presenting a challenge to understanding how quickly and through what mechanisms glaciers such as Thwaites can retreat from sites of sea-floor stabilization.

One place where information on patterns and processes of retreat might be studied is in former ice-sheet grounding zones. Offshore of Thwaites Glacier, recent sonar mapping documented numerous sea-bed promontories where the ice was previously grounded^18^. Spectral analyses of this former bed topography indicate that these regions are geologically analogous to the modern grounding zone^18^, making them ideal sites to target the processes and behaviour of Thwaites in the recent past.

As present-day retreat partly represents adjustments to past imbalance^19^, it is important to constrain how glaciers such as Thwaites changed before observations were possible. Direct observations of grounding-line change and forcing extend back only 30–40 years for most of West Antarctica, and although ice-sheet retreat following the Last Glacial Maximum is mostly well constrained, data relating to the past 10,000 years of Thwaites Glacier history are scarce^20^. Substantial gaps therefore remain in our understanding of grounding-line behaviour in the pre-satellite era.

A recent detailed study of grounding-zone wedges offshore of the Antarctic Peninsula, formed during the last deglaciation, revealed delicate grounding-line landforms that record exceptionally fast rates of past retreat^21^ demonstrating potential to resolve subannual rates of change from submarine landforms. However, Antarctic deglaciation at the time of this retreat was driven in part by sea-level rise from the melting of the Northern Hemisphere ice sheets^22^, meaning neither the time period nor forcing provide appropriate analogues for the near-future state of the West Antarctic Ice Sheet. In addition, although satellite monitoring since the 1990s has substantially improved the temporal resolution at which small-scale grounding zone changes can now be sensed^2,23^, so far, there has been no way of reconstructing rates of past grounding-line retreat over annual or even subannual, let alone daily, time frames, for historical time periods (for example, pre-1990s) that might further improve simulations of future West Antarctic Ice Sheet evolution.

In this Article, to address vital questions about rates and processes of recent grounding-line retreat, we studied an isolated sea-floor promontory (the ‘bump’) at the southwest corner of the remnant TGT, downstream of what is now Thwaites Glacier’s fastest-flowing grounded section^7^ (Fig. 1a). The bump, at ~630–670 m water depth, is deeper than the keel depths of modern icebergs or ice shelf drafts^24^ so post-retreat processes are unlikely to have disturbed landforms produced during grounding-zone retreat (Fig. 1b). The site lies within a broader tributary trough (T2 in Fig. 1) that is today a flow path for modified Circumpolar Deep Water (CDW) that circulates into and out of the ice-shelf cavity^25^. The bump is flanked to the east and west by two narrow subtroughs at approximately 800 m depth (Fig. 1d). We deployed an autonomous underwater vehicle (AUV) mounted with geophysical sensors to acquire high-resolution (sub-metre) multibeam bathymetry and acoustic imagery from sidescan sonar, flying at an altitude of 50–90 m from the sea floor. Approximately 13 km^2^ of new geophysical data were obtained over a 19 h mission across the bump (Fig. 1c).

## Observations from a former West Antarctic grounding zone

**Fig. 2:**
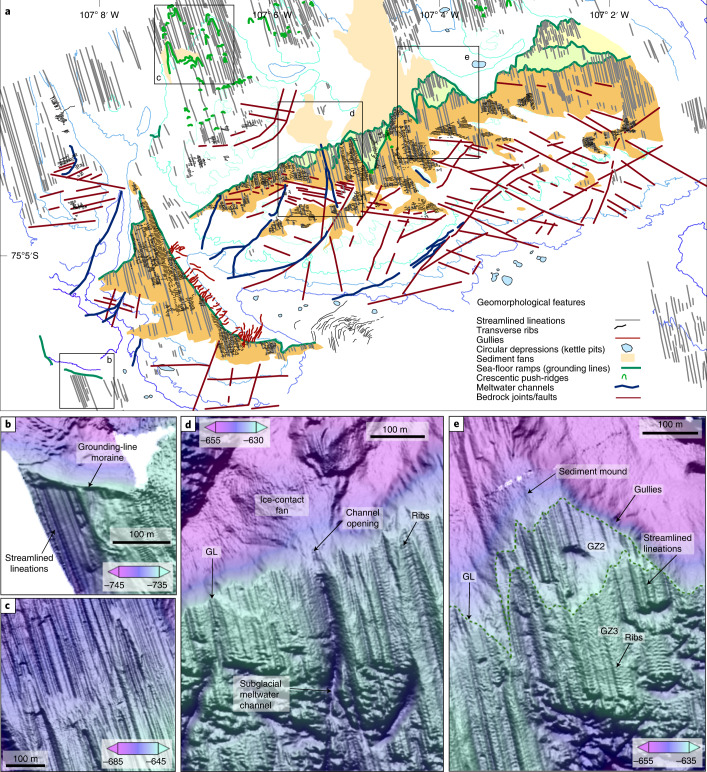
Sedimentary grounding zone system mapped on ‘the bump’ seaward of Thwaites ice shelf. **a**, Geomorphological map of features on and seaward of the bump. Coloured regions are successive sedimentary tops of former grounding zones (GZ 1–3). **b**, AUV multibeam bathymetry showing a grounding-line moraine marking the edge of a flat-topped lineated ramp. **c**, Examples of connecting crescentic push-ridges formed on downward-dipping slopes in the northern part of the study area. **d**, Subglacial meltwater channel and a connected ice-contact fan system. **e**, Examples of multiple back-stepping grounding lines (small ramps: green, dashed lines), lineations and overprinting ribs shown in more detail in subsequent figures.

Sea-floor imagery reveals that the bump is a former grounding zone of Thwaites Glacier (Fig. 1d and Supplementary Fig. 1). From geomorphological mapping of the main promontories and northern half of the survey, we identify landforms that characterize a sedimentary grounding zone system^26^ (Fig. 2). This sedimentary landscape is contrasted to the south and on smaller up-ice slopes of sea-floor highs by exposed and heavily jointed bedrock (Figs. 1d and 2a, Supplementary Fig. 2 and Supplementary Text). Three former grounding-zone fronts are identifiable, traceable as tortuous, steep, 0.5–2 m-high ramps that can be mapped west to east, in some cases for approximately 2 km (Fig. 2a). The ramps are leading edges of till sheets laid down by grounded ice in contact with the sea floor, each marking successive steps of ice-sheet retreat southwards (Fig. 2a, GZ1–3; Supplementary Fig. 3). Straight and parallel sets of subglacial lineations characterize the sea bed between the ramps, becoming younger landward. The most extensive grounding zone (Fig. 2a,e, ‘GZ3’) is also the most recent, meaning the sea-bed record on top of the bump mainly relates to a single grounding period.

Multiple linear channels that shoal northwards cut through the lineated surfaces (Fig. 2a). One of these—a 6 m deep and 75 m wide channel—terminates in an ice-proximal fan, clearly showing the emergence of a subglacial meltwater conduit at the former grounding line (Fig. 2d and Supplementary Fig. 4). Gullies on slopes that coincide with former grounding-line positions, fans and meltwater features, together, indicate that Thwaites Glacier was fully grounded through its present-day ice-shelf cavity and possessed an active sediment and subglacial meltwater system when the grounding line retreated onto the bump, leaving landforms preserved as the glacier receded.

**Fig. 3:**
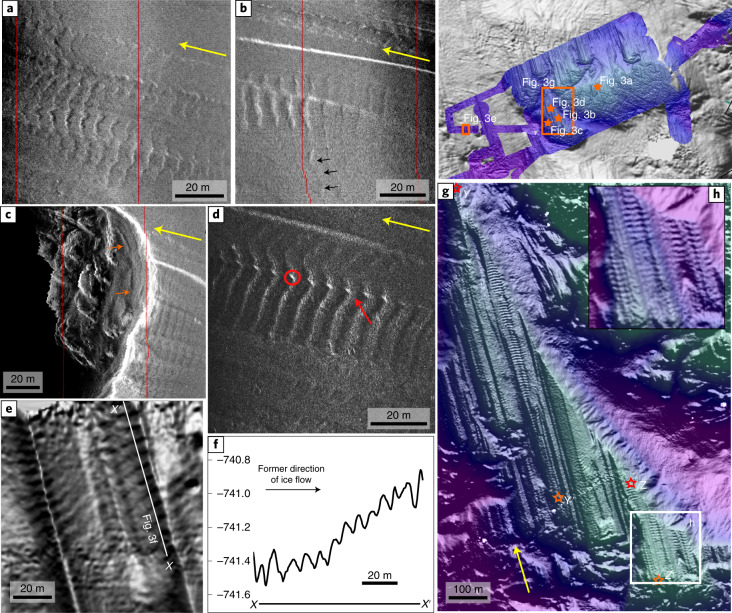
Details of sea-floor ribs on high-resolution AUV sidescan and multibeam data. **a**–**d**, Examples of high-frequency sidescan imagery illustrating the back-stepping conformity of ridge shape (**a**), non-alignment of ribs to underlying lineations (**b**), rib formation on terraces (**c**) and the ‘beading’ (red circle) and overprinting (red arrow) of existing subglacial features (**d**). **e**, Multibeam hillshade showing fine-scale landforms, <20 cm high, crossing lineation ridges and grooves. **f**, Corresponding profile *X*–*X*′, demonstrating the subtle geometries of some of the landforms (5–20 cm) and their surprising depth (>740 m). **g**,**h**, Multibeam swath bathymetry covering the longest series of ribs (profile *Y*–*Y*′ and *Z*–*Z*′ combined; stars mark start and end of profile sections). Inset shows close-up example of lateral continuity in the southern portion of the ribs. Black arrows in ‘b’ mark lateral continuation of one oblique ridge. Yellow arrows in each image show ice flow direction inferred from lineations.

On seaward dipping, shallow (~660 m) portions of the sea bed, high-frequency sidescan data reveal tracks of parallel ribs transverse to former ice flow direction (Fig. 3a–d). The ribs relate dominantly to the most recent grounding surface, GZ3 (Fig. 2a). They are similar to regularly spaced ‘rungs’ recently observed in the Weddell Sea^21^. Ribs overprint and are traceable across multiple underlying lineations and form ‘beaded’ features at lineation ridge crests where they obliterate the pre-existing subglacial signature (Fig. 3d and Supplementary Fig. 7). Some ridges sit obliquely across the underlying lineations, distinguishing them from the processes that created the subglacial bedforms (Fig. 3b). The ribs are subtle—70% of them are <20 cm in height—but widespread through the study region (*n* > 1,500; Fig. 2a).

**Fig. 4:**
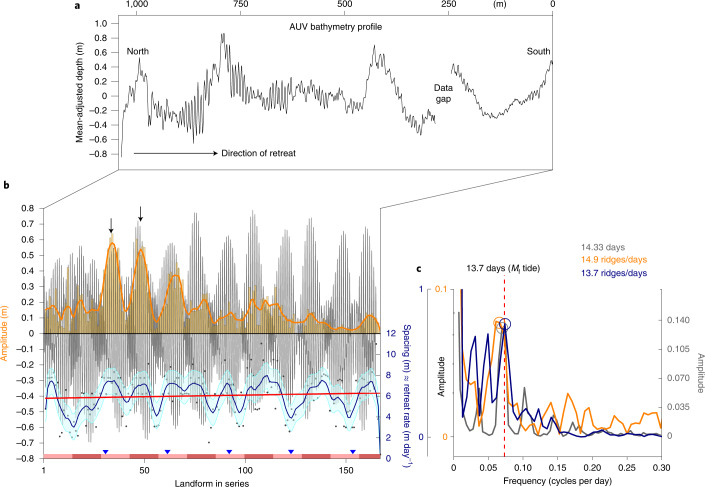
Geometries and analysis of the longest series of ribs from the top of the bump. **a**, Mean-adjusted composite topographic profile through the longest series of sea-floor ridges (see profile Y-Z’ in Fig. 3h for location). **b**, Extracted geometries (Methods) from **a** showing the longitudinal variability in height (orange) and spacing (blue) of 164 ribs plotted together with the output from the tide model^28^ (black; Methods). Alternating pink bars are 14-day periods, with blue triangles marking monthly intervals in the series (30.4 days). **c**, Periodograms (fast Fourier transforms) of the datasets in **b** showing the significance of frequencies in the series (cycles per day). Coloured circles mark the dominant peaks in the frequency spectra of the tidal model (grey), ridge amplitude data (orange) and ridge spacing data (blue), respectively. The tidal periodicity of 14.33 days in the tide model is the recurrence interval for spring and neap tides due to the interaction of principal lunar and principal solar semidiurnal tides. Red dashed line marks the *M*_f_ (lunar fortnightly) constituent.

To assess the processes that formed the ridges, we analysed the geometry of the longest series of ribs comprising 164 individual landforms (Figs. 2e, 3g and 4a). Amplitudes of the 0.1–0.7 m high ribs show clear along-flow modulation, with a 13–15 ridge periodicity repeating over ten cycles (Fig. 4b). Spectral analysis of the along-flow variance in rib heights shows a dominant frequency peak at 14.9 ridges (Fig. 4c, orange). The overall peak amplitude of each cycle decreases gradually southwards. The periodicity is not unique to one series and is preserved in adjacent sequences of ribs found at different depths. This cyclic variability is mirrored in the spacing of the ribs along flow, and the two parameters co-vary (Fig. 4b and Supplementary Fig. 8). Ten cycles occur over the 1,050 m long profile. Between 0 m and 420 m along profile, the peak interval is about 21–23 ridges. From 420 m to 1,050 m along profile, peak intervals range from 12 to 16 ridges. The dominant period is calculated at 13.7 ridges (Fig. 4c, blue). The largest spacing between ridges correlates with higher peaks in ridge amplitude, showing that, generally, ribs are further apart when the ribs are taller (Supplementary Fig. 8d). Rib spacing is typically 6.6–7.6 m but ranges overall from ~1.6 m to 10.5 m (Supplementary Fig. 8b).

The amplitude, spacing and the 13–15 cycle amplitude variation match with present-day tides in this region^27^ (Fig. 4 and Methods) that are predominantly diurnal (that is, one high and one low tide per day). The amplitude reaches ±0.8 m, and there is a pronounced 14-day spring–neap cycle (grey, Fig. 4b). This suggests that tides modulate the ridges in both amplitude and spacing (Figs. 4b,c, and Supplementary Fig. 8), and that the ribs are imprints of a feature at the bottom of the ice (such as in the grounding zone). When plotted together with the tide model predictions for the Thwaites region^28,29^ (Methods), there is a strong coherence between the variability in the amplitudes of the ribs and daily tidal heights (Fig. 4b and Supplementary Fig. 8; *R*^2^ = 0.57). The 13–15 cycle periodicity is particularly clear in the peak-to-peak spacing and amplitude in the northern and central parts of the profile (arrowed, Fig. 4b; Fig. 4c). The spatial sequence of ribs therefore represents a series in time, and diurnal and spring–neap tidal constituents are the strongest influence over their geometry (Fig. 4c).

## Ribs formed by tidally modulated grounding-line retreat

**Fig. 5:**
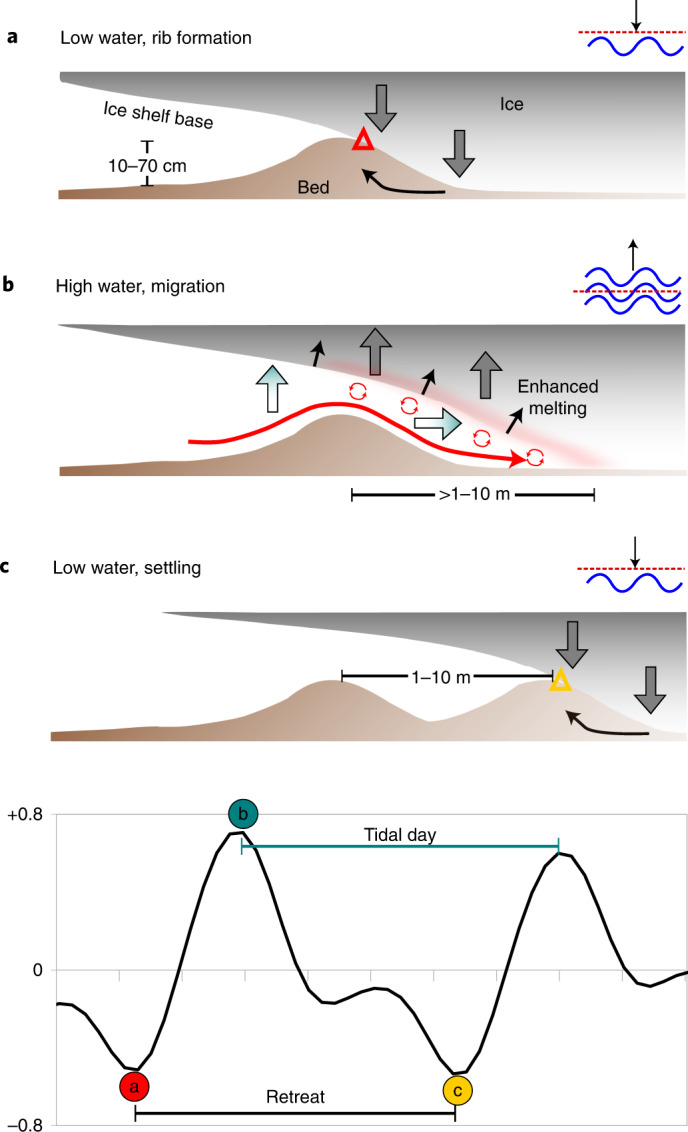
Conceptual model for the formation of ribs by tidally modulated grounding-line migration. **a**, Grounding at low water forms a rib by settling and sediment bulging/extrusion at the grounding line ahead of an ice plain. **b**, Subsequent grounding-line migration occurs during high water with the ice-shelf base displaced upwards and landwards by the tide, allowing contact with warm ocean water. Basal melting is enhanced by tidal mixing in the narrow cavity. **c**, Grounding line settles at new position on the next low water, creating a second rib in series. The amplitude and spacing of ribs is a function of tidal amplitude, which modulates the distance of retreat from ridge to ridge. Bottom: plot depicting typical diurnal tide cycle for a 48 h period at Thwaites Glacier. Intervals on x-axis correspond to 0.2 days (4.8 hours). **a**–**c** relate to positions on the tidal cycle. Triangles denote successive grounding-line positions. Note exaggerated vertical scale in all images; the true sea-floor expression of the ridges is subtle, with sea bed and ice bottom slopes only fractions of a degree.

What process could create the distinct ribs? Forming in series, one per day, the ribs could relate either to the forward motion by grounded ice (that is, ice shelf/ice mélange keels^30^) or to its inland migration (Supplementary Information). Tidal flexure and fast, steady retreat of the grounding line—first lifting during high water, and then settling on the sea bed during low tide—can best explain the unusual rib features (Fig. 5; cf. ref. ^21^). Sediment, extruded each time the grounding line settles on its substrate, forms a chain of ridges in the narrow accommodation space of the sub-ice shelf cavity (Fig. 5). The shape, regularity and periodicity of the ribs imply vertical displacement at the grounding zone with grounding-line retreat proceeding at many metres per day.

The present study provides a rare example where the clear influence of tides has left geological markers of their interaction with the ice sheet on the sea bed (Fig. 4b)^31^. The rapid retreat documented by the ribs suggests an ice-dynamic response, but ocean-forced melting may also have played a key role. On the basis of the common amplitude of the ribs (approximately 0.1–0.4 m) and assuming daily rates of recession of 6–8 m per day, we estimate the daily ice loss rate to exceed 1,000 kg per day per metre of the grounding zone (Supplementary Information and Supplementary Fig. 11). A heat flux exceeding 600 W m^−^^2^ is required to melt that quantity of ice. This compares to the 300–900 W m^−^^2^ that can be delivered to the ice by ~1.2 °C water (3.1 °C above in situ freezing point), if there is a fully turbulent ice–ocean boundary layer, and to the 0.9 TW (or 300 W m^−^^2^ for an ice shelf with an area of 3,000 km^2^) that is delivered through one of the deep troughs leading into the cavity from the north^25^. The distances between sequential ribs (Fig. 4b) show grounding-line migration ranged from ~4 m per day during neap tides to ~8 m per day during spring tides, and an explanation for this variation may be the increased turbulence during spring tides^32^ (Supplementary Information).

Ribs are not found everywhere despite the grounding line having retreated through the study area. We suggest that low angle bed and ice slopes (that is, low gradients in height above buoyancy) are essential for the ribs to form. This configuration favours rapid migration of the grounding line, and low topographic variability allows ridges with a ‘pure’ tidal signal to be deposited sequentially. Regions of low-gradient ice surface and bed are ice plains; weakly grounded regions of ice streams immediately upstream of their grounding lines with low basal shear stress and small elevations above buoyancy^33^. An important implication is that when Thwaites Glacier grounding line sits on such high points even small amounts of ice-shelf thinning could instigate phases of rapid retreat.

## Pulses of rapid retreat at Thwaites Glacier

A second major implication of the formation mechanism is that the spacing of the ribs enables reconstruction of the rate of grounding-line recession of Thwaites Glacier in the past. The average spacing of the rib series increases upstream from 5.8 m to 6.3 m (Fig. 4b, red line). Converted to retreat rate, this corresponds to an 8% increase with time. Over a 5.5 month period, Thwaites Glacier retreated across the bump at an accelerated rate: from 2.12 km per year to 2.3 km per year. It has been suggested^9^ that retreat from Thwaites Glacier’s offshore pinning points, across the deep inner cavity to its present-day position, was probably rapid. Our results indicate that the rate of retreat from the bump was double the average estimated for the period 1996 to 2009, and about three times faster than a location immediately inland of the bump between 2011 and 2017 (0.6–0.8 km per year (ref. ^7^)). The majority of observations for Amundsen Sea glaciers covering the past 30 years have shown retreat rates to not exceed 1–2 km per year^34^. For example, the Smith/Kohler Glaciers retreated 35 km between 1992 and 2011 at an average rate of approximately 1.8 km per year^2^, while Smith Glacier West retreated at a rate of 2 km per year between 2016 and 2018^34^. These rates—lower than those recorded on the bump—underscore the exceptionally fast retreat phase uncovered by our data. One notable study at nearby Pope Glacier observed almost 4 km of grounding-line retreat over a period of just under 4 months in 2017^34^, equivalent to a retreat rate >11 km per year. Taken together with our results, long-term retreat rates obtained by satellites or airborne geophysical missions (on the order of 0.35–1 km per year over decades^7^) and derived from the offshore geological record (approximately 0.01–0.2 km per year over millennia^35^) may, therefore, hide temporal variability in grounding-line behaviour, including rapid, punctuated retreat phases that exceed the typically documented upper bounds on the pace of West Antarctic Ice Sheet recession.

Fast retreat from flat-topped ridges may be a process that is already occurring in several places at Thwaites Glacier today^9^. In previous work, the process of sediment removal from offshore sea-bed highs was suggested to have made the Thwaites Ice Shelf more sensitive to thinning in the past by lowering sea-bed topography^18^. Our results show that, in addition to increasing bathymetric depth, the process of flattening sea-bed highs during grounding may have also increased the likelihood of rapid retreat at Thwaites Glacier by promoting the development of ice plains at which rapid inland tidal migration of the grounding line occurred. Erosion from the tops of analogous highs on which the grounding line sits today may have broadened the modern grounding zone. In doing so, this process may expose wider regions of intermittently grounded ice to melting through delivery of oceanic heat through a narrow cavity, thereby increasing the sensitivity of Thwaites Glacier to tidal forcing (Fig. 5b). Coupled ice–ocean finite element models of Thwaites Glacier have highlighted that wider grounding zones are more susceptible to faster rates of retreat^16^. Thus, we suggest that bed modification by erosion at the base of streaming ice may have pre-conditioned Thwaites Glacier to rapid recession. Moreover, in the presence of relatively unchanging external forcing (that is, persistent basal melting), non-linear phases of fast retreat can be expected as a result of fundamental grounding-zone processes.

Establishing the timing of retreat across the bump and, as a consequence, the age of the ribs on its surface remains to be resolved by direct sampling. The ice shelf was intact above the bump until the past decade^11^, and as recently as 1992 and 2011, TGT was ephemerally in contact with a pinning point, at shallow depths of approximately 300 m^2^. In airborne radar data from 1978, the grounding line was already situated southward of the bump^36^. Assuming current retreat rates of 0.8 km per year persisted back through time, and a monotonic retreat from the bump to the present-day grounding line, the ribs almost certainly pre-date the 1950s, and may be several centuries old if a slower average rate of retreat is invoked (for example, they would be approximately 180 years old, if the retreat rate was 0.3 km per year^7^.

Irrespective of the exact age of the landforms, the results shown here form a direct analogue for the glacier’s current grounding zone, which is situated on elevated sea-floor ridges at similar water depths to the bump. Our landform evidence showing rapid retreat from a sea-bed ridge adjacent to the ice shelf suggests that major changes in flow dynamics could be initiated within months should the grounding line start to detach from high points in the sub-ice landscape.

The data described in this paper are unique in several aspects. They provide a rare example where the influence of tides is clear and has left imprints on the sea bed. The rates of retreat inferred from the landforms resolve daily grounding line motion for a key West Antarctic ice stream over nearly half a year, from a time period in which observations were not possible. We show one of probably many pulses of rapid retreat that characterized Thwaites Glacier’s inland migration where the ice lost contact with topographic stabilizing highs. Rapid thinning and retreat will shorten the recurrence interval between such events, and in the context of recent observations, thinning and progressive grounding-line retreat at Thwaites Glacier increases the probability of such a pulse occurring in coming decades. The challenge for models predicting ice-sheet evolution is to now replicate the precise sequence of grounding-line movements across the bump, and to include processes of tidal migration and ice-plain formation in their physics. By evaluating models against our new high-resolution palaeo-data, it will be possible to gain a better understanding of Thwaites Glacier’s ongoing retreat trajectory and its contributions to sea-level rise, which could threaten coastal communities and ecosystems in the next few human lifetimes.

## Methods

### Data acquisition and processing

Cruise NBP19-02 took place between January and March 2019 on the United States Antarctic Program icebreaking vessel RV *Nathaniel B. Palmer* as part of the wider National Science Foundation–Natural Environment Research Council funded International Thwaites Glacier Collaboration involving UK, US and multi-national affiliates. Onboard, we collected underway multibeam swath bathymetry, sub-bottom profiler data and recovered marine sediment cores to improve our understanding of Thwaites Glacier history. For this paper, we focus on datasets collected by the deployment of a 6,000-m-depth-rated free-swimming Kongsberg HUGIN AUV. The AUV, operated by the Swedish Research Council and maintained by the University of Gothenburg, was trialled and underwent first deployments in polar waters during the cruise. ‘Ran’ is equipped with a variety of oceanographic sensors, as well as a full suite of geophysical instrumentation. Here we show data collected using an AUV-mounted single-head Kongsberg EM2040 multibeam echo-sounder and a dual-frequency Edgetech 2205 sidescan sonar. Both sonars enabled the collection of high-resolution images of the sea bed at a resolution unrivalled for shelf regions around the West Antarctic Ice Sheet.

AUV mission 009 deployed the HUGIN at 75° 04.21′ S, 106° 58.89′ W, at 22:07h on 28 February 2019. Recovery occurred at 15:33h on 1 March 2019 at 75° 05.409′ S, 107° 10.925′ W. The total mission time was 17 h 26 min. Navigation was achieved by coupling an onboard INS (inertial navigation system consisting of accelerometers and gyros) with two 12 kHz Universal Transponder Positioning (UTP; cNODES) units deployed at known locations that served as call and response range-positioning for the vehicle. In addition, the vehicle operated using a Doppler velocity log whereby it tracked the sea floor or sea surface via dead reckoning. Acquisition heights varied during the survey between 50 m and 95 m. Processing of navigation data was undertaken onboard in Kongsberg proprietary NavLab software. However, owing to noise from the first UTP station, the filtered navigation required further processing subsequent to the cruise by Kongsberg engineers in Norway.

Processing of the AUV multibeam swath bathymetry data using a first-pass cleaned navigation was undertaken in MB system while onboard the *Palmer*. The steps involved (1) extraction of navigational and attitude data, (2) bathymetry data conversion, (3) ping editing to remove spurious soundings and (4) gridding. Because of the varied flying height, an onboard gridded dataset was produced at a conservative 1.5 m grid cell size, which formed the basis of most bathymetric analysis in this paper. We gridded using a Gaussian-weighted mean algorithm in the mbgrid program, adopting a spline interpolation to fill grid cells not filled by swath data for gaps up to six grid cells in size. The grid was exported in an ESRI ASCII format for manipulation in a geographic information system. The 1.5 m grid was used for the majority of observations made in our analyses.

Post-processing of the AUV data by Kongsberg technicians removed noise problems associated with the first of the two UTPs that hampered processing of the vehicle’s navigation onboard. In post-processing, we removed fixes from the first UTP entirely. Consequently, for the start of the mission, the AUV ran on dead reckoning with GPS fix until the second UTP was encountered after which navigation was determined by UTP-INS. A cleaned and filtered real-time navigation using a best-fit solution was output via NavLab. Loops (avoidance turns) in the vehicle track, of which there were several, were removed, and a static shift of 95 m at 130° was applied. A second grid with improved resolution of 0.7 m was produced for subsequent visualization and verification of our analyses from the coarser, 1.5 m gridded dataset.

Images of the sea floor acquired by the AUV’s sidescan sonar were replayed and exported using Kongsberg reflection post-acquisition visualization software, isolating the high-frequency channel (400 kHz). Each image was slant-range corrected and processed with a pixel size of 0.05 × 0.05 m.

### Geomorphological mapping of landforms

Glacial landforms were mapped from the gridded multibeam datasets in a geographic information system using associated hillshade surfaces and corresponding sidescan sonar imagery for guidance. All mapping was undertaken at horizontal scales between 1:500 and 1:5,000. We used the original 1.5 m multibeam grid for the majority of feature mapping but updated the map with subsequent mapping work in selected areas using digitized landforms from the later post-processed 70 cm grid. Any updates to the map at this second stage were geo-located to be consistent with the original mapping using an *XY* shift. As the landform map was generated before secondary post-processing of the AUV data, it should be noted that the completed map has a small *XY* offset of approximately 95 m at 130°. However, the data as a whole are internally consistent, meaning the interpretations and subsequent geometric analysis of the ribs are not subject to any appreciable change. Landforms were classified by geometry, independent of their genetic interpretation following best practices described in a variety of glacial geomorphological literature. Extended descriptions of the landforms and their genetic classification are included in Supplementary Section A2.

To extract landform statistics from the bathymetric data, we employed a semi-automated picking approach. Profiles were extracted from the bathymetry along the sets of ribs of interest, sampled at a horizontal resolution of 10 cm. The extracted long profiles were detrended and mean-adjusted by removing a least-squares regression from the profile. Subsequently, the data were filtered by subtracting a 100-point adjacent averaging smooth of the detrended data, which served to remove long-wavelength features in the bathymetry profile while retaining the finer details and geometry of smaller-scale landforms of interest. From the filtered and detrended data, a manual baseline was digitized that assigned nodes to the troughs between ribs and interpolated between them. We subtracted the baseline from the data so that the ribs in series were levelled to a zero horizon. A peak analysis tool was then used to identify rib crests in the processed profiles from which height from baseline (amplitude) and peak-to-peak spacing were automatically derived.

### Tidal model

To investigate the potential role of tides in forming sea-floor landforms, we extracted predicted tidal amplitudes from a tidal model of the global ocean for a location seaward of the Thwaites Glacier front (108° W, 74° S). This was carried out to provide a handle on the expected modern-day tidal amplitudes at the ice-shelf margin and to establish the dominant tidal constituents driving ice-shelf and grounding-line flexure on both short and long timescales. We used the Oregon State University TPXO-9 Global Tidal Model for analysis^28^. The model includes complex amplitudes of relative sea-surface elevations for eight primary (M2, S2, N2, K2, K1, O1, P1 and Q1), two long-period (Mf and Mm) and five non-linear (M4, MS4, MN4, 2N2 and S1) harmonic constituents.

The time window chosen for comparison covered the period 22 March 2020 to 4 September 2020 and was sampled at hourly intervals encompassing nearly 12 spring–neap cycles (166 days on plotted axis in Fig. 4). The dates selected for the tidal record comparison began at the end of the austral summer, and were forecast through to the end of the following austral spring. The late March start date for the time series reflects an attempt to correlate the landform record with a time period in which West Antarctica is generally at its warmest, and sea ice is at a minimum in the Amundsen Sea. Although investigations of the seasonal variability in the properties of CDW in the easternmost Amundsen Sea embayment generally indicate a thicker and slightly warmer deep water layer during winter^38^, we view the end of summer as the most likely time interval in which rapid ice sheet retreat events might be triggered. Modelling studies indicate that the largest southward transport of heat and the thickest CDW layer peaks in the March–May period. For guiding our choice, other rapid changes in Antarctic glaciers have typically been observed to have initiated in the late summer period: for example, the Larsen A Ice Shelf disintegrated in January 1995, while Larsen B collapsed during the period 31 January to March 2002, and Wilkins Ice Shelf underwent partial collapse through February and March of 2008. Whereas collapses of the Antarctic Peninsula ice shelves have mainly been triggered by surface melting and hydrofracture as a result of atmospheric warmth, changes at Thwaites Glacier are likely to be ocean or sea-level instigated. Although there is no explicit link between the timing of major calving events and grounding-zone retreat, we note observations of major changes in Thwaites Glacier in March of previous years: Thwaites had a partial outburst of its western mélange in the period immediately following our AUV survey in early March 2019, while the large iceberg B-22 previously calved from the Thwaites Ice Tongue in mid-March 2002.

### Analysis of rib landforms

The periodograms in Fig. 4 of the main manuscript investigate the dominant periods (frequencies) in the series of ribs compared with the frequency content of the tidal series. The dominant frequencies describe important periodicities in the data. The periodograms were produced using a fast Fourier transform implemented in OriginLab. Frequency corresponds to cycles per unit of time. In this case, the unit of time was set to 1 calendar day so that periods equate to 1/peak frequency. For the tidal model, we filtered the hourly modelled tidal amplitude outputs from the TPXO-9 model using a 24-point percentile filter with a percentile value set at 99 (Supplementary Fig. 13). This corresponded to replacing the signal point with the 99th percentile value of the data points in the moving data window. The resultant filtered dataset removes the 24, 12 and 6 h tidal periodicities that dominate the full-resolution tidal dataset (Supplementary Fig. 13b), preserving and tracking closely the daily peak tidal height. We compared this approach with a method that decimated the data to a single time per day. Both achieve similar results in emphasizing the dominance of a fortnightly spring–neap cyclicity in the daily tidal values.

For the ribs, we analysed the dominant frequencies in the amplitude data as a series. Since the amplitude data are already detrended and extracted from the initial topography, they represent an appropriate way of investigating periods in the data. However, periodograms of the filtered and mean-adjusted topography produce results with no discernible differences. In each FFT (Fast Fourier Transform) analysis, we carried out the routine using a Hanning window and display the amplitude as a function of frequency.

## Online content

Any methods, additional references, Nature Research reporting summaries, source data, extended data, supplementary information, acknowledgements, peer review information; details of author contributions and competing interests; and statements of data and code availability are available at 10.1038/s41561-022-01019-9.

## Supplementary information

Supplementary Information Supplementary text (site description, extended technical descriptions and interpretations of landforms, extended description of rib formation, timing of rib formation, and oceanic forcing of grounding-line retreat) and Figs. 1–13.

## Data Availability

All data needed to evaluate the conclusions in the paper are present in the paper and/or Supplementary Information. A high-resolution multibeam raster grid from AUV mission 009, on which the majority of the analyses in this paper were based, is available for download from the PANGAEA database (https://pangaea.de) and figshare (10.6084/m9.figshare.20359920).
