# Supplementary material for: Rapid retreat of Thwaites Glacier in the pre-satellite era
Source: Nat Geosci. 2022 Sep 5;15(9):706–13. doi: 10.1038/s41561-022-01019-9 (PMC12602364; doi:10.1038/s41561-022-01019-9)
Supplement: Supplementary file 1 — Supplementary text (site description, extended technical descriptions and interpretations of landforms, extended description of rib formation, timing of rib formation, and oceanic forcing of grounding-line retreat) and Figs. 1–13. [file 41561_2022_1019_MOESM1_ESM.pdf]

nature portfolio

https://doi.org/10.1038/s41561-022-01019-9

Supplementary information

Rapid retreat of Thwaites Glacier in the pre-satellite era

In the format provided by the
authors and unedited

Supplementary Information for

Rapid retreat of Thwaites Glacier in the pre-satellite era

Alastair G.C. Graham, Anna Wåhlin, Kelly A. Hogan, Frank O. Nitsche, Karen J. Heywood, Rebecca L. Totten, James A. Smith, Claus-Dieter Hillenbrand, Lauren M. Simkins, John B. Anderson, Julia S. Wellner, Robert D. Larter

Correspondence to: alastairg@usf.edu

This file includes:

Supplementary Text
- A1. Site description
- A2. Extended technical descriptions and interpretations of landforms
- A3. Extended description of rib formation
- A4. Timing of rib formation
- A5. Oceanic forcing of grounding line retreat

Supplementary Figs. S1 to S13

Supplementary Text

A1. Site Description

Thwaites Glacier, alongside its neighboring Pine Island Glacier, is one of the main ice streams draining into the southern Amundsen Sea embayment. The seaward extension of the Thwaites Glacier ice-sheet bed is a 1200 m deep NE-SW oriented trough – the Thwaites Trough - along which former ice flow would have discharged. During the Last Glacial Maximum, the Pine Island and Thwaites systems were convergent as a single, major paleo-ice stream[1]. The stepped retreat of the ice stream, as well as the sea-floor imprint it left behind, has been well documented [2,3]. However, the inner continental shelf of the Amundsen Sea embayment has been poorly mapped until recently[4], especially in the ocean in front of Thwaites Glacier which is prone to heavy and persistent sea ice cover.

The 'bump' is a newly mapped sea-floor pinnacle at the southwest corner of the Thwaites Glacier Tongue. The bump consists of a flat-topped pinnacle with a smooth gently-sloping distal flank, and a rugged, steeper proximal slope. The feature is c. 2800 m wide and 1500 m long. Water depths across the bump range from 670–630 m, shoaling gradually towards the west of the feature.

A2. Extended technical descriptions and interpretations of landforms

At the broadest scale, the sea bed in our study area can be sub-divided descriptively into two substrate regimes; one comprising of exposed bedrock, the other consisting of a sedimentary bottom that is, in many cases, likely to be a veneer of sediment deposited over bedrock undulations. This sediment thickness is not known but we estimate that it varies between 1 to many 10s of meters thick, rather than consisting of 100s of metres of sediment that typify basin fills in other continental margin settings. The basement that forms the underlying basis for the large-scale regional topography is thought to consist of Paleozoic to Mesozoic granites which form a batholith belt related to the Gondwana active margin[5]. The granite is overlain in the modern subglacial environment by Cenozoic volcanics but is largely exposed at the sea floor in the offshore setting.

Sea-floor highs occur in chains or belts that possess a strong southwest-northeast fabric in the vicinity of Thwaites Glacier. The highs are likely structural features that are related to rifting during the Late Mesozoic, and which formed as part of the establishment and evolution of the West Antarctic Rift System[6]. Intervening glacially eroded troughs have a similar orientation to the chains of highs suggesting they are partly structurally controlled as well, at least on the inner shelf near to Thwaites Glacier. At 1200 m the Thwaites Trough is the largest and deepest glacial trough at the sea bed near to our study site. Several deep channels connect at 90 degrees to the trough, one of which contains the 'bump' surveyed in this study. The overall geomorphological context for the bump was reported by Hogan et al.[4].

On maps of roughness and in sub-surface acoustic profiles from the region, the troughs are generally smoother, sedimented regions of the sea floor. Thwaites Trough in particular is an area

of glacial and postglacial sediment accumulation[4]. Small sub-basins and sub-troughs also frequently contain drapes or fills of sediment 10s of meters thick. In contrast, rougher slopes and highs are generally covered by only thin veneers of sediment, or sediment is absent.

The detailed geomorphological landscape of the sea floor on and around the bump is complex, representing many different glacial geological processes (Fig. S1). Bedrock joints and scars are extensively distributed at the sea-floor in the Amundsen Sea where they make up a large part of the geological fabric, especially in inner shelf locations where the basement rocks are exposed or only thinly sediment-covered. In the vicinity of the bump, we mapped clear bedrock fabric on the heads of crags, and extensively on the backslope of the bump (Fig. S2). The fabric of the terrain is complex and not the immediate focus of our study. However, some of the larger and more continuous elongated ramps or valleys are likely to be syn-rift faults. There are also conjugate faults that are oblique to the main west-east trending structural elements.

The importance of bedrock in shaping the overall terrain is exemplified in the rock-cored crags that have been the seed points for the down-flow deposition of sediment under the former flow of grounded glacial ice. Crag-and-tails are characteristic landforms of fast-flowing warm-based ice sheets. They consist of eroded rock heads with elongated sedimentary tails that taper downstream. The rock-cored heads are typically comprised of more-resistant substrate, which in the case of Thwaites geology, is consistent with the presence of granitic highs at the sea bed. The flow of ice around the head creates a cavity in the lee of the bedrock protuberance and an associated high pressure gradient[7]. Till flows from high to low pressure regions of the subglacial bed, infilling the cavity with sediment. The cavity and the tail can propagate along-flow under fast ice velocities and with a ready supply of sediment, the elongation of which is countered only by the rate at which the cavity can close via processes of creep.

Five crag and tails were mapped in our study area (Fig. S2). They are 200-400 m wide and a maximum of 15-40-m high. Bedrock heads give way to sedimentary tails that are themselves superimposed by multiple smaller and more subtle lineations. The presence of crag and tails provides good evidence that basal ice was at melting point and that sliding has been an important subglacial mechanism in the development of some of the sea-bed landforms at the Thwaites grounding zone. Under the modern Thwaites Glacier, radar profiles show that analogous bedrock bumps are present which have hard stoss sides and softer lees, consistent with a bedrock crag and till-formed tail interpretation[8,9]. This suggests that the geomorphology of the bump and its surrounding features is representative of the geomorphic landscapes and processes that exist and operate under the modern-day Thwaites Glacier system.

Mega-scale glacial lineations (MSGLs) are highly-elongated, parallel, ridge-groove landforms that are common signatures of ice-sheet beds[10,11]. Although originally described on satellite images from terrestrial regions of the former Laurentide ice sheet, MSGLs have since been more commonly described as well-preserved sea-bed landform assemblages on the relict beds of former ice streams that drained ice caps and ice sheets, in the Northern Hemisphere, Greenland, the sub-Antarctic, and Antarctica. Critically, using radar techniques, lineations have been imaged under several active Antarctic ice streams which possess evolving, lubricated, and weak subglacial deforming beds[12,13]. They are therefore, unequivocally, the direct imprints of fast ice stream flow over sedimentary substrates. Typical MSGL sizes are on the order of 1->10 km, with

peak-to-peak spacing centered around c. 300 m, and elongations generally greater than 10:1[11]. However, the lower end of the size ranges reported for such MSGL may be truncated by poor preservation and a lack of imaging techniques that can define ice-stream bed geomorphology at the fine scale[14].

In our AUV data, streamlined lineations are interpreted as MSGLs (e.g. Fig. S3). They are usually a few tens up to >500 m long, spaced between 20-50-m from peak to peak, and have amplitudes in the region of 0.2-2 m, but they are typically 1-1.5 m high. Their high elongation ratios, and their parallel streamlined geometries are consistent with an ice-contact basal signature created by a process linked to subglacial deformation (such as an instability at the interface of the ice stream and a weak, deformable underlying till). Notably, these landforms are smaller than typical MSGLs reported from other glaciated margins and surveyed using conventional shipborne sonar techniques. Davies *et al.* reported similar finer-scale lineations adjacent to larger wavelength bedforms on the top of the Jenkins Ridge underneath Pine Island Glacier ice shelf using comparable sonar technology deployed on an AUV[15]. Interestingly, they interpreted the smaller scale of bedforms as a product of lightly-grounded ice stream flow; an interpretation that is consistent with our wider interpretation of the 'bump' existing as a past ice plain environment in which the coupling between ice and the underlying bed is weak and intermittent. MSGLs at this scale, as reported here, occur across the top of the Bump sea-floor highs but also superimpose the seaward flanks (tails) of the large crag-and-tails described previously.

Landforms interpreted to have formed by meltwater erosion are a key part of the geomorphological landscape at Thwaites Glacier ice front (Figs. S2, S4). Linear channels are mapped across the bump, with several terminating at the northern edge of the flat upper surface. They are distinct from underlying structural geological fabric from observations that they cut through terrain that is superimposed by sediments, are deeply incised compared to other linear faults and joints in the bedrock, and maintain a channel structure throughout their entire length. One channel in particular is highlighted in the main paper and shown in more detail in Figure S4. The 5-9 m-deep channel is c. 500 m long, has an undulating thalweg and v-shaped cross profile along its length. It connects a subglacial meltwater conduit to a distal feature that we interpret as an ice-contact fan formed seaward of the grounding line[16]. The fan is a maximum of 220 by 270 m in size, and up to 7 m thick based on profile extrapolations from adjacent topography. The meltwater fan was likely deposited at the same time that the channel was active, and because we can show that the channel is a relatively recent feature, we infer that the fan must also be a product of recent grounding line retreat and sedimentation.

Our observations indicate that the flow of water at the grounding line was likely organized through a set of eroded canals rather than via a distributed through-till or sheet system. Previous work on the modern bed signature of subglacial meltwater discovered that substantial water volumes are ponding in a system of distributed canals upstream of a subglacial bedrock ridge that is breached, and that these canals transition downstream into a system of concentrated upwardly-incised water-filled channels[17]. The continued transition of this meltwater downstream is not known, but our data support the notion that water reaching the Thwaites grounding zone remains focused in channel systems and actively erodes the bed in the process, delivering substantial quantities of sediment to the grounding line. Other authors have speculated that through reducing basal drag, meltwater might also play an important role in lubricating the bed,

reducing basal shear stress, and facilitating faster ice flow and retreat. There is no evidence from our dataset that meltwater has been responsible for initiating or accelerating the pace of retreat at Thwaites; an observation that has also been verified in the modern Thwaites glacier system using measurements from satellite data[18,19]. However, from the sequence of landforms formed in the study area (i.e. channel-fan systems cut across the lineated landscape so were formed immediately prior to grounding line retreat), it seems likely that meltwater channels were active immediately prior to the grounding-line retreat recorded by the ribs. Furthermore, in our analysis, there is no evidence that the emergence of meltwater at point sources has influenced the geometry of the past grounding line by reducing sediment supply to it, as has been hypothesized in other studies from relict ice sheet beds[20]. If anything, we make the opposite assertion by showing an association of ice-proximal deposits with a distinct isolated channel outlet.

Other ambiguous features of the terrain that are ascribed to meltwater processes are shown in Figure S5. They are mapped out in Figures 2 and S2 as depressions and include circular potholes as well as linked concentric features that may represent proto-channels. The circular landforms measure 10–80 m wide, 0.8–7 m deep, and have broadly u-shaped profiles, while the channels they sometimes form are 70 to 650 m long. They are geographically restricted to the deeper portions of the study site, found in excess of 700 m water depth, on the southern and western flanks of the bump and in intervening sub-troughs. The features seem to be formed exclusively in sedimentary substrates based on the smoother appearance of the ocean floor in their vicinity.

The landforms are best explained by the breaching of the subglacial environment by large-scale meltwater flows. The isolated nature of the landforms suggests that the features are not a direct product of persistent melt from warm-based ice, but rather have formed by outbursts at discrete times and in discrete locations at the ice-sheet bed. Moulins delivering surface water to the bed of the ice sheet are one possible mechanism for routing water to produce circular melt potholes[21]. For this process to be viable, liquid water in excess of pore space saturation in the glacier firn is needed in order to form ponds and this, in turn, requires significant surface melting on the ice sheet. However, the existence and persistence of surface melt ponds at Thwaites is debatable. Surface melt only occurs sporadically in the Amundsen Sea today. In addition, the widespread existence of surface drainage at Thwaites has not been well documented although recent research into Antarctic-wide surface hydrology has provided good evidence for surface melt pools on a number of marine-terminating glaciers through the late 20th Century, including the neighboring Pine Island ice shelf[22]. Furthermore, short-lived surface melt events have been detected at Thwaites Glacier using satellite passive microwave imagery of the ice sheet surface[23], suggesting that warming coupled with specific meteorological conditions could be favorable to surface melting. Any such surface melt events however, are too short in duration to produce significant quantities of meltwater to form surface pools and moulins. Therefore, any meltwater signatures are likely a record of past activity during times of elevated melt supply. Ice core records from West Antarctica show extreme positive temperature anomalies in the 1936–45 decade, associated partially with a pronounced El Nino event[24]; the last time the Amundsen Sea experienced significant warming above the overall pattern of 20th Century change. During this time, the Pine Island Glacier ice shelf underwent a major phase of retreat[25] which was also accompanied by a strengthening of CDW inflow onto the Amundsen Sea continental shelf[26]. It is tempting to speculate that the record of grounding zone changes on the Bump, including the generation of

surface meltwater at Thwaites Glacier, and the erosion of subglacial meltwater potholes, could be linked to the same mid-20th Century climatological episode.

Alternatively, melt from subglacial lakes trapped upstream of the bump[18], is a possible source of the meltwater necessary to form the depressions and channel systems, although the basal routing of subglacial meltwater does not easily explain the production of isolated potholes in the glacier bed. As such, these meltwater features remain open to interpretation and the focus of further research.

Grounding zone wedges, represented by terrace-like 'ramps' in the bathymetry, characterize the northern edge and surface of the bump (Fig. S3). These are reminiscent of similar ice marginal features mapped in a variety of relict settings, and that have been hypothesized to be actively forming at modern grounding lines[27,28]. These features are interpreted consistently as the sedimentary depositional product of grounding line stability, marking either the edge of an extensive subglacial till 'sheet'[28] or a wedge shaped accumulation of subglacial sediments at the ice sheet edge[29]. When found preserved and overprinting one another in sets, they are usually the product of step-wise grounding line retreat. Although larger wedges may take centuries or even millennia to form, it is plausible that smaller ramps and ice-marginal moraines may be created on annual or even sub-annual timeframes. In this study, we interpret the ramps as landforms of retreat in accordance with many other studies, and this is supported by the cross-profile and plan-shape geometry of the features as well as the relationship of other landforms (gullies and lineations that initiate and terminate respectively at the ramp fronts) to the ramps. The ramps are 0.2-2 m high, and continuous for up to several kilometers.

On the deeper streamlined flanks of the tails of the crags, we image grounded pushed-up ridges at the distal ends of ploughed grooves (Main manuscript, Figs 2A, 2C). These possess similar geometries to ice-ploughed ridges mapped in other formerly-glaciated environments, but are clearly formed in a downhill direction (Fig. S6). They can therefore only have formed at the grounding line, as irregularities at the ice-bed interface transitioned into sub-ice shelf keels. Mapped in clusters adjacent to one another, we interpret the ridges as pseudo-grounding lines marking retreat from positions when Thwaites Glacier was extended beyond the bump (Fig 2A).

Grounding-line push ridges are less well known than grounding zone wedges. Indeed, we suggest these are a new class of landform revealed by the high resolution of our AUV dataset and have, to our knowledge, not been described before in sediment grounding zone systems (Fig. S6). The features typically comprise of crescent-shaped ridges 0.2-1 m high, and 10-25 m long, spanning either the width of only a single lineation or sometimes forming a semi-continuous ridge that is significantly wider than a single furrow. In the latter, the ridges combine to form moraine-like ridges. The seaward slope of the ridges is lower profile than the ice-proximal side and is notably smooth. They also clearly overprint lineations and other subglacial signatures that lie underneath the crescentic ridges. In profile, and as observed in plan-view, the ridges have deeper trailing furrows suggesting these are ploughed features. The landforms are similar to ice-keel pushed ridges found on glaciated margins elsewhere[30], except that in those cases the ridges are always formed on shoaling sea-floors and can thus be easily explained by the grounding of floating ice keels (either from iceberg keels or the irregular underside of floating ice shelves/mélange). In the Thwaites Glacier data, we interpret the ridges as the depositional

product of keels ploughed into the sediment by the forward advancement of grounded ice. The only feasible way to produce these ridges, in contrast to floating ice analogues, is if the keels existed in the grounding zone of the grounded glacier and were created as the ice reached flotation and ungrounded from its forward-sloping seabed. Some of these ridges can be traced laterally and form west-east linear chains that mimic grounding line shapes. We thus suggest the ridges are pseudo-grounding line wedges. They must form during relatively rapid retreat of the grounding line across the sea bed and show relatively asymmetric retreat between sites of local grounding and unpinning. Stable periods of grounding line recession would likely lead to more continuous lateral moraines or wedges. This indicates that ice retreat may have been relatively rapid before the grounding zone established itself on the bump. However, we do not have additional information on when or how quickly ice retreat proceeded through this zone of the sea floor. Additional surveys northward of the bump through the Thwaites proximal marine realm may reveal further landform suites that uncover the broader progression of ice retreat from the Holocene through to the present day.

Finally, in addition to pushed grounding zone features, in the sub-trough south of the bump the sea bed is characterized by a number of small arcing ridges. These have the appearance of more traditional moraine landforms that typically form at tidewater glacier margins. They have an arcuate morphology consistent with a local lobe of the grounding line retreating through the deep. They remain the subject of further interrogation.

A3. Extended description of rib formation

Forming in series, one per day, the ribs could relate either to the forward motion by grounded ice (i.e. ice shelf/ice mélange keels) or to its landward retreat. Several aspects of the AUV data display similarities with observations of tidally-forced velocities in Antarctic ice streams. For example, the mean spacing of ridges matches well to velocities of c. 7–8 m per day measured across the Thwaites Glacier Tongue grounding line for the period 2014-2018.

Four lines of evidence, however, are inconsistent with the ridges having formed by keels under an intact floating ice shelf or by the forward movement of keels grounded in an ice mélange. First, the ribs are found over large areas and at a number of water depths, including those >740 m (Fig. 3E, 3F), which is not consistent with generation by keels beneath floating ice (either icebergs or ice shelves). Second, some of the ribs occur obliquely to the orientation of underlying lineations which would not be the case should keels be responsible for the generation of both landforms (Fig. 3b); indeed, the 'beading' of lineations beneath the ribs demonstrates that the ribs cross-cut already pre-existing signatures of subglacial origin (Fig. S7). This observation differs from those in other areas in which similar ribbed 'corrugations' have been mapped, where the ridges formed concurrently with the grooves of ice-ploughed scours[2]. Third, although there is a preferred rib spacing of c. 7 m, the range of variability is surprisingly large. Tidal stress transmission related to tidal amplitude has been shown to influence surface glacier flow velocity by 10–20% at Rutford Ice Stream, and by a factor of 3 on Ice Stream D[31]. However, the absolute changes in day-to-day velocity amount to several tenths of a meter at most and cannot explain forward motion that can vary one day to another by many meters. We would therefore expect a more uniform spacing of ridges if the ribs were formed daily by ice shelf flow overriding the bump. Larger velocity variations might be more consistent with the motion of an ice mélange.

However, profiles of the sea bed on which ribs are formed dip seaward away from the glacier (Fig. S9) and the ribs climb and descend larger wavelength bumps in the sea bed. Furthermore, the area is likely too deep (>600-800 m) to contain a partly grounded ice mélange. All of these observations allow us to reject a floating ice hypothesis for the ribs, ruling out formation under an ice shelf and by the keels of a floating ice mélange. In addition, the ribs show no preferred asymmetry in either direction (Fig. S10) so are neither push moraines formed by substantial readvances (e.g. during winter; which would show a steep distal slope;[32]) nor grounding line moraines formed by till advection (which would show a steep proximal slope).

Ribs similar to those described in this study have been noted from surveys of the sea bed in glacial environments for a number of decades. Observations from sidescan sonar during expeditions to the Weddell Sea in the mid-1970's provided the first images of regularly spaced ridges at the sea bed, referred to in subsequent studies as 'washboard moraines', and interpreted as the result of the tidal 'wobble' of icebergs grounding on the sea bed[33]. Shipp et al. (1999) observed near-identical washboard patterns in the Ross Sea using sidescan sonar and inferred a similar mechanism for their formation[34]. This same model was taken up and modified in studies that later undertook bathymetric surveys of the middle shelf in the Amundsen Sea embayment, West Antarctica. Using more sophisticated shipborne multibeam sonar, Jakobsson et al. (2011) mapped large areas of subtle 'corrugation ridges' that retain a tidal modulation in their amplitude along flow that is very similar to the tidal cyclicity we have discovered offshore of Thwaites Glacier in this study (i.e. spring-neap dominant)[2]. At only a few meters high but 10s to c. 200 m apart, the authors interpreted the ridges as the result of past ice shelf collapses that calved armadas of tall icebergs into the coastal ocean encased in a proglacial ice mélange. The trailing keels of these icebergs subsequently grounded intermittently during low tide as they travelled seaward and encountered a shoaling sea floor. In another study of the sea bed underneath Pine Island Ice Shelf, corrugations spaced apart at distances of 40-120 m were mapped from the *Autosub-3* AUV on the flat top of 'Jenkins Ridge'[35]. Under an extant ice shelf that to our knowledge has not receded landward of its present-day calving front position in the last c. 10,000 years, an iceberg hypothesis was deemed unlikely for these corrugation ridges. Instead, in that study, Graham et al. (2013) interpreted the ridges as the result of ice keel interaction with the sea bed during intermittent ice-shelf grounding.

Do all corrugation ridges form in the same way? The answer to the question based on previous interpretations of corrugation formation appears to be no. There is little doubt that some corrugation ridges form at the trailing end of icebergs subject to tidal motion. For example, they have been mapped inside of the incised curvi-linear tracks of iceberg ploughmarks, in some cases far from any known grounding line environment, past or present[36]. However, the recent discovery of 'ribs' on the backslope of grounding zone wedges in the Larsen Shelf region of Antarctica also provides convincing evidence that similar bedforms can be created at the grounding line of ice sheets as they recede. The spacing of ridges requires extremely high rates of retreat if, as the authors suggest, the ridges are formed daily or twice-daily by the action of tides lifting and settling at the grounding zone. In previous studies, this rapid retreat appeared implausible and has been one obstacle to interpreting corrugation ridges as grounding line features. Another is that, until now, we have neither been able to image the ridges at the level of detail necessary to be sure of their geometries nor have we had images of corrugation ridges with

a clear glacial geomorphological context that has allowed us to deduce the details of their formational processes[37].

The formation of ribs in this study agrees with the basic model described by Dowdeswell et al. (2020) who also referred to the landforms as ‘rung and ladder’ features. We retain the use of ‘ribs’ here as a descriptor. The differences in terminology are arbitrary given that (1) there is no formalized nomenclature yet for the landforms, (2) neither is there any clear agreement on how the features form, and (3) moreover, it is very likely that geometrically-similar bedforms can be created in a range of different ways, in different environments under the specific conditions that (a) the ice is close to buoyancy, (b) is subject to a substantial tidal forcing, and (c) has a vector that allows for motion in one direction; either a continual phase of advance (as in iceberg drift) or a continual phase of retreat (as in grounding line recession). We distinguish the ribs from ‘ribbed moraines’, found in cold-based zones of ice sheets near to their former divides[38,39], which we view as entirely separate in terms of their morphology, genesis, and paleoglaciological implications.

We expand upon the description of the physical mechanism put forward by Dowdeswell et al. (2020) by inferring that ribs form only under certain glaciological situations (namely, in ice plains), and also centralize the combined role of dynamic thinning and ocean melting, when coupled to tides, in causing melt variability that influences the pace of grounding line retreat.

The ribs are interpreted to have formed as a product of interaction of the grounding zone with an existing sedimentary substrate. We do not envisage that the ribs were emplaced in their entirety and directly by deposition of till advected to the grounding line because this would require a laterally continuous and very rapid flux of material, as well as extremely high rates of sediment comminution in order to sustain till supply in order to extrude and construct a single rib every c. 12 hours. Rather, we suggest the ribs were formed by the compressive action of the settling grounding line during low tide, which led to the extrusion of a pre-existing deformable till bed that had been laid down during prior glacier advance. The ribs can therefore be considered as solemarks of the grounding zone environment (akin to squeeze moraines formed at tidewater margins by glaciotectonic processes) rather than depositional subglacial bedforms in the traditional glacial geological sense (e.g. dump or end moraines). Whatever the process that forms ribs, the action of the grounding line interacting with the sediment bed is sufficient to overcome (i.e. has a greater effect on the geomorphic imprint than) any direct deposition by the supply of subglacial till.

We find no evidence for pairing in the ribs as has been recorded and hypothesized in previous work[40], but can attribute this lack of signal to the dominance of the diurnal tides over Thwaites ice shelf dynamics that exists today (Figs. S8, S12, S13). We can infer that a similar tidal regime existed in the historical past and that semi-diurnal tides were not influential on the Thwaites vicinity over previous centuries. In some parts of the grounding zone, it is also clear that the tidal modulation is dampened during retreat, emphasized by tracks of ribs, particularly towards the eastern part of the study area, in which the amplitudes and spacings do not retain as clear a signal of tidal modulation as features on the western portion of the bump.

One question we wished to address in relation to rib formation concerned the role of inflowing and outflowing water at the grounding zone - a process that is likely to occur in many ice stream grounding zone environments[41,42] and which may be occurring at Thwaites grounding zone too - in generating or obscuring tidal ribs. Tidal pumping effects may be strong during the settling of the grounding line on the sediment substrate, especially during spring tides. There is potential that the expulsion of water and sediment is particularly high during this phase and for erosive currents to be generated through the drawing in and pumping of entrained seawater. However, under ice plain conditions, where the grounding zone is only lightly grounded, pressure gradients that could generate erosive flows are likely to be weaker. Furthermore, there is no evidence in the dataset for the erosion of ribbed geomorphology. This may be due to the fact that the ribs are very low profile features that do not present significant obstacles to the flow or pathway of water or fine sediment during the ejection of water from the grounding line. In addition, we can show cycles of ribs that are clearly unbroken in sequence and have therefore not experienced any significant modification by proglacial processes.

A recent study by Warburton et al.[43] linked the rise and fall of tides to the movement of water in the subglacial environment. The authors show an asymmetry between the fast movement of water into the grounding zone during the incoming tide, and the retention and slower drainage of ocean water from the grounding zone as the tide recedes. Crucially for our study, the model shows that if the drainage of subglacial water is fast, with barely any drag exerted by the hydrological system, then the grounding line can move up and down freely with diurnal tides. However, gradual reduction in permeability filters the effect of tides so that the fortnightly tidal component becomes more dominant. Eventually if the drainage of subglacial water is very slow, because of low permeability through the till, the grounding line becomes fixed at the high tide position, disconnecting the response of the grounded zone from the ocean tides.

These modelling cases suggest that for Thwaites Glacier to have formed the ribs in our study, a well drained and/or high permeability grounding zone is necessary with very little retention of inflowing ocean water in the grounding zone environment. Because permeability of tills in the grounding zone is likely to be low, we envisage networks of small drainage channels cut into the till to be the likely mechanism by which water evacuates the grounding zone effectively. Discontinuities in the geological substrate, such as along exposed sea-bed fault structures and through local substrate depressions, might have provided alternative pathways for water to drain away from the non-till based regions of the grounding zone. Although we have interpreted a purely extrusional mechanism for the ribs due to tidally-forced rising and settling of the grounding zone, further investigation and testing of rib formation or modification under the influence of ‘estuarine’ flow of water in the grounding zone should be the attention of continued research into rib phenomena.

<u>A4. Timing of rib formation</u>

In the absence of direct sampling on the bump itself, the chronological constraints we present in the main are our best estimates of the age of the rib sequence. The nearest dated sediment core recording grounding line retreat that we can have confidence in is PS75/167 reported by Hillenbrand et al.[26] The sediments in that core show a minimum grounding line retreat date of 10,350 cal. ka BP (with 11 dates from shallower in the core that lie between approximately 10

and 8 ka). However, the site is ~20 km north of the tip of the Thwaites Eastern Ice Shelf and ~60 km from the area described in this paper so do not provide indicators for grounding line location or age that could further refine our estimates for retreat from the bump.

Retreat rates through the Holocene were much slower than encountered in the Amundsen Sea today – c. 10 m per year[44], whereas retreat over the past 30 years has been on average 0.5 to 1 km/yr based on observations from satellite data[45]. Furthermore, the recent rates of accelerated retreat probably developed sometime after the mid-20th century[46]. Therefore, given the proximity of the bump to the modern ice margin, it is reasonable and appropriate to extrapolate ages using a conservative rate of retreat as recorded by recent observations (using the modern grounding line position as a point of reference) rather than utilizing early Holocene ages from cores distal to the study site. Our best estimates taking this approach suggest that retreat from the bump occurred at some point in the last 180 years. This interpretation is supported by Tinto et al.[47] who speculated that Thwaites Glacier was fully grounded through its cavity on shallow sea bed highs between c. 50 and 150 years ago, and subsequently retreated rapidly. At neighbouring Pine Island Glacier, rapid retreat from a sea bed high situated underneath the floating ice shelf only started during the 1940s. Until we have data to show otherwise, a reasonable assumption is that Thwaites Glacier followed a similar pattern of change. This inference is further supported by observations from sub-bottom profiler data collected by the AUV on top of the sea-floor high, which show the absence of an acoustically-laminated postglacial drape that commonly covers deglacial sedimentary sequences on the Antarctic continental shelf. The lack of any substantial sediment cover provides additional support for the features on the surface of the bump having been exposed by ice retreat in recent decades or centuries rather than many millennia ago.

Other marine sediment cores collected on cruise NBP19-02 currently do not refine our estimates for retreat from the bump because they are located in distal parts of the Thwaites inner shelf region and do not capture the necessary sequence of sedimentary facies to show grounding line variability in recent times.

A5. Oceanic forcing of grounding line retreat

A5.1. Estimate of basal melt from trough amplitude and distance between troughs

In the main text, we described the potential role of oceanic forcing on grounding line retreat. Here we expand upon the physical processes occurring at the grounding zone in order for ribs to be formed; specifically, we show the requirements for steady ice mass loss (likely a large component of which is attributable to dynamic thinning) to coincide with tidal modulation of melt patterns in the grounding zone in order to explain adequately the appearance of the rib landscape. In the following, we also consider the case in which ocean-forced melting is responsible for the entirety of the mass loss (with no ice shelf thinning) showing, in the process, that ocean-driven processes are capable of explaining the ribs and their patterns mapped in this study.

Consider an ice base near the grounding zone (Fig. S11). The ice retreats at a steady pace (red arrows), while the ice sheet simultaneously moves up and down with the tides (green arrows).

The minimum ice volume V_{min} that has been lost between two consecutive high tides over the stretch W is hence given by:

$$V_{min} = a \cdot W \cdot \lambda, \qquad (1)$$

where a is the amplitude of the tide oscillation and λ is the distance between two troughs. This ice is lost in 24 hours, giving a melt rate M (kg/s) of:

$$M = \frac{\rho_{ICE} V_{min}}{24h}, \qquad (2)$$

where ρ_{ICE} (kg/m^3) is the density of the ice. Using the amplitude a = 0.2 m, wavelength λ = 6 m and ρ_{ICE} = 900 kg/m^3 gives a melt rate of about 1080 kg/day per m of grounding zone. The energy E_{ICE} required to melt this volume is given by

$$E_{ICE} = L \cdot M \qquad (3)$$

where L is the latent heat of melting for ice. Using L = 334 kJ/kg gives E_{ICE} = 361 10^6 J per day per m of grounding zone, or the heat flux H_F (W/m^2)

$$H_F = E_{ICE}/W/\lambda, \qquad (4)$$

which (using M = 1080*W kg/day, 86 400 s in a day, and λ = 6 m) gives H_F = 696 W/m^2.

A5.2. Heat flux through boundary layer

Assuming a fully turbulent boundary layer, and that the ice base temperature is equal to the freezing temperature, the ablation rate a_b (m/s) can be parameterized as[48]

$$a_b = \frac{\rho_w}{\rho_i}\frac{C_P}{L} u_* \gamma_T (T_W - T_F) \qquad (5)$$

where ρ_w and ρ_i (kg/m^3) is the density of water and ice, respectively, C_P (J/kg/K) is the specific heat capacity of water, L (J/kg) is the latent heat of melting, u_* (m/s) is the friction velocity, T_W is the in situ water temperature, T_F is the freezing temperature, and γ_T is the turbulent transfer coefficient. The transfer coefficient varies depending on the boundary layer characteristics[49], and obtains values between 0.001 to 0.05 for Antarctic ice shelves[48,49]. Using (5) an ablation rate between 30 and 80 m/year (depending on friction velocity) was obtained for 1.2 ^oC warm water[49], corresponding to a heat flux of between 317 and 847 W/m^2. The friction velocity at the ice-ocean interface is given by[49]

$$u_*^2 = C_D U^2, \qquad (6)$$

where $C_D = 1.5 \cdot 10^{-3}$ is the drag coefficient and U is the velocity outside the boundary layer. Assuming only the tides cause movement in the water beneath the ice shelf and using γ_T = 0.002, TW = 1.2 ^oC and the tide velocities from the CATs2008 tide model[50] the parameterization (5) gives the amplitude, velocity and ablation rate shown in Fig. S12 for a two-month cycle. The tide

velocity at coordinates 75° S and 107° W were calculated with the tide_pred algorithm[50] and used in (5) and (6) to parameterize the basal melt rate.

Based on the results shown in Fig. S12 the ablation rate would increase from its minimum value near zero during neap tides to its maximum value 1.5 cm/day during spring tides. However, in reality the tides only make up a portion of the velocity at the ice-ocean boundary. A model study of the Amundsen Sea ice shelves[48] indicate that the average basal melt rate at the present-day Thwaites ice shelf is increased by 7.8% due to tides. This means that the background velocity and turbulence, induced by buoyant meltwater near the ice-ocean boundary, accounts for over 92.2% of the total melt. If the same ratio is true for the location studied here, it would mean that the background melt rate is about 4.6 cm/day and it increases to 6 cm/day during spring tides. Ice shelves that are further away from the 'critical latitude' (i.e. the latitude at which the inertial frequency equals the tidal frequency, 74° 28' S for the M2 constituent[51]) are expected to have larger tide-induced portions of basal melt[51]. The tide models of today are based on present geometry of ice and seabed, and it is not expected that the numbers obtained here are necessarily correct for the time period when the ice retreated from the bump. The numbers and estimates should rather be seen as a reality check for the mechanisms proposed to cause the formation of tidal ribs at the sea bed. Further investigation of the dual roles of dynamic thinning and basal melt variability are warranted.

A5.3. Water needs to be replenished in order to melt the ice

The heat available to melt ice in water of temperature T ^{o}C is given by

$$E_W = C_P \rho_W (T - T_F) \cdot h \cdot \lambda \,, \qquad (7)$$

where C_P is the heat capacity of water, ρ_W is the density of water, T_F is the freezing point of sea water, and h is the thickness of the water column. Using C_P = 4200 J/K/kg, ρ_W = 1000 kg/m^3, T_F = -1.9 ^{o}C, T = 1.2 ^{o}C, h = 1 m and λ = 6 m gives E = $8 \cdot 10^6$ J per m of grounding zone, i.e. only about 2.5% of the energy needed each day to melt ice. This indicates that, in the process of creating ribs, the warm water needs to be replenished several times during one day in order to melt the ice, which in turn requires an inflow rate at around 1 mm/s or larger.

Supplementary References

1. A.G.C., G. *et al.* Flow and retreat of the late Quaternary Pine Island–Thwaites palaeo-ice stream, West Antarctica. *J. Geophys. Res.* **115**,.
2. Jakobsson, M. *et al.* Geological record of ice shelf break-up and grounding line retreat, Pine Island Bay, West Antarctica. *Geology* **39**, 691–694 (2011).
3. Kirshner, A. E. *et al.* Post-LGM deglaciation in Pine Island Bay, West Antarctica. *Quat. Sci. Rev.* **38**, 11–26 (2012).
4. Hogan, K. A. *et al.* Revealing the former bed of Thwaites Glacier using sea-floor bathymetry. *Cryosph. Discuss.* **2020**, 1–36 (2020).

5. Kipf, A. *et al.* Granitoids and dykes of the Pine Island Bay region, West Antarctica. *Antarct. Sci.* **24**, 473–484 (2012).

6. Gohl, K. Basement control on past ice sheet dynamics in the Amundsen Sea Embayment, West Antarctica. *Palaeogeogr. Palaeoclimatol. Palaeoecol.* **335**–**336**, 35–41 (2012).

7. Benn, D. I. Fluted moraine formation and till genesis below a temperate valley glacier: Slettmarkbreen, Jotunheimen, southern Norway. *Sedimentology* **41**, 279–292 (1994).

8. Clyne, E. R., Anandakrishnan, S., Muto, A., Alley, R. B. & Voigt, D. E. Interpretation of topography and bed properties beneath Thwaites Glacier, West Antarctica using seismic reflection methods. *Earth Planet. Sci. Lett.* **550**, (2020).

9. Holschuh, N., Christianson, K., Paden, J., Alley, R. B. & Anandakrishnan, S. Linking postglacial landscapes to glacier dynamics using swath radar at Thwaites glacier, Antarctica. *Geology* **48**, 268–272 (2020).

10. Clark, C. D. Mega-scale glacial lineations and cross-cutting ice-flow landforms. *Earth Surf. Process. Landforms* **18**, 1–29 (1993).

11. Spagnolo, M. Size, shape and spatial arrangement of mega-scale glacial lineations from a large and diverse dataset. *Earth Surf. Proc. L.* **39**, 1432–1448 (2014).

12. Bingham, R. G. *et al.* Diverse landscapes beneath Pine Island Glacier influence ice flow. *Nat. Commun.* **8**, (2017).

13. King, E. C., Hindmarsh, R. C. A. & Stokes, C. R. Formation of mega-scale glacial lineations observed beneath a West Antarctic ice stream. *Nat. Geosci.* **2**, 585–588 (2009).

14. Clark, C. D. Emergent drumlins and their clones: From till dilatancy to flow instabilities. *Journal of Glaciology* vol. 56 1011–1025 (2011).

15. Davies, D. *et al.* High-resolution sub-ice-shelf seafloor records of twentieth century ungrounding and retreat of Pine Island Glacier, West Antarctica. *J. Geophys. Res. Earth Surf.* **122**, 1698–1714 (2017).

16. Dowdeswell, J. A. *et al.* Sediment-rich meltwater plumes and ice-proximal fans at the margins of modern and ancient tidewater glaciers: Observations and modeling. *Sedimentology* **62**, 1665–1692 (2015).

17. Schroeder, D. M., Blankenship, D. D. & Young, D. A. Evidence for a water system transition beneath Thwaites Glacier, West Antarctica. *Proc. Natl. Acad. Sci. U. S. A.* **110**, 12225–8 (2013).

18. Smith, B. E., Gourmelen, N., Huth, A. & Joughin, I. Connected subglacial lake drainage beneath Thwaites Glacier, West Antarctica. *Cryosphere* **11**, 451–467 (2017).

19. Hoffman, A. O., Christianson, K., Shapero, D., Smith, B. E. & Joughin, I. Brief communication: Heterogenous thinning and subglacial lake activity on Thwaites Glacier, West Antarctica. *Cryosphere* **14**, 4603–4609 (2020).

20. Simkins, L. M. *et al.* Anatomy of a meltwater drainage system beneath the ancestral East Antarctic ice sheet. *Nat. Geosci.* **10**, 691–697 (2017).

21. Jantunen, T. The spatial distribution of potholes in Uusimaa, southern Finland. *Bull. Geol. Soc. Finl.* **68**, 40–45 (1996).

22. Kingslake, J., Ely, J. C., Das, I. & Bell, R. E. Widespread movement of meltwater onto and across Antarctic ice shelves. *Nature* **544**, 349–352 (2017).

23. Ghiz, M. *et al.* Energetics of Surface Melt in West Antarctica. *Cryosph. Discuss.* 1–42 (2020) doi:10.5194/tc-2020-311.

24. Schneider, D. P. & Steig, E. J. Ice cores record significant 1940s Antarctic warmth related to tropical climate variability. *Proc. Natl. Acad. Sci. U. S. A.* **105**, 12154–12158 (2008).

25. Smith, J. A. *et al.* Sub-ice-shelf sediments record history of twentieth-century retreat of Pine Island Glacier. *Nature* **541**, 77–80 (2017).

26. Hillenbrand, C. West Antarctic Ice Sheet retreat driven by Holocene warm water incursions. *Nature* **547**, 43–48 (2017).

27. Anandakrishnan, S., Catania, G. A., Alley, R. B. & Horgan, H. J. Discovery of till deposition at the grounding line of Whillans Ice Stream. *Science (80-.).* **315**, 1835–1838 (2007).

28. Larter, R. D. & Vanneste, L. E. Relict subglacial deltas on the Antarctic Peninsula outer shelf. *Geology* **23**, 33–36 (1995).

29. Dowdeswell, J. A. & Fugelli, E. M. G. The seismic architecture and geometry of grounding-zone wedges formed at the marine margins of past ice sheets. *Bull. Geol. Soc. Am.* **124**, 1750–1761 (2012).

30. Gales, J. A., Larter, R. D. & Leat, P. T. Iceberg ploughmarks and associated sediment ridges on the southern Weddell Sea margin. *Geol. Soc. London, Mem.* **46**, 289–290 (2016).

31. Anandakrishnan, S., Voigt, D. E., Alley, R. B. & King, M. A. Ice stream D flow speed is strongly modulated by the tide beneath the Ross Ice Shelf. *Geophys. Res. Lett.* **30**, (2003).

32. Ottesen, D. & Dowdeswell, J. A. Assemblages of submarine landforms produced by tidewater glaciers in Svalbard. *J. Geophys. Res.* **111**, F01016 (2006).

33. Barnes, P. . & Lien, R. Iceberg rework shelf sediments to 500 m off Antarctic. *Geology* **16**, 1130–1133 (1988).

34. Shipp, S. Late Pleistocene-Holocene retreat of the West Antarctic Ice-Sheet system in the Ross Sea: Part 1—Geophysical results. *Geol. Soc. Am. Bull.* **111**, 1486–1516 (1999).

35. Graham, A. G. C. *et al.* Seabed corrugations beneath an Antarctic ice shelf revealed by autonomous underwater vehicle survey: Origin and implications for the history of Pine Island Glacier. *J. Geophys. Res. Earth Surf.* **118**, 1356–1366 (2013).

36. Hall, B. L. & Denton, G. H. Radiocarbon chronology of Ross Sea drift, eastern Taylor Valley, Antarctica: Evidence for a grounded ice sheet in the Ross Sea at the last glacial maximum. *Geogr. Ann. Ser. A Phys. Geogr.* **82**, 305–336 (2000).

37. Batchelor, C. L. *et al.* New insights into the formation of submarine glacial landforms from high-resolution Autonomous Underwater Vehicle data. *Geomorphology* **370**, 107396 (2020).

38. Dunlop, P. & Clark, C. D. The morphological characteristics of ribbed moraine. *Quat. Sci. Rev.* **25**, 1668–1691 (2006).

39. Lindén, M., Möller, P. & Adrielsson, L. Ribbed moraine formed by subglacial folding, thrust stacking and lee-side cavity infill. *Boreas* **37**, 102–131 (2008).

40. Dowdeswell, J. *et al.* Delicate seafloor landforms reveal past Antarctic grounding-line retreat of kilometers per year. (2020).

41. Horgan, H. J. *et al.* Estuaries beneath ice sheets. *Geology* **41**, 1159–1162 (2013).

42. Walker, R. T. *et al.* Ice-shelf tidal flexure and subglacial pressure variations. *Earth Planet. Sci. Lett.* **361**, 422–428.

43. Warburton, K. L. P., Hewitt, D. R. & Neufeld, J. A. Tidal Grounding-Line Migration Modulated by Subglacial Hydrology. *Geophys. Res. Lett.* **47**, (2020).

44. Smith, J. New constraints on the timing of West Antarctic Ice Sheet retreat in the eastern Amundsen Sea since the Last Glacial Maximum. *Glob. Planet. Chang.* **122**, 224–237 (2014).

45. Milillo, P. *et al.* Heterogeneous retreat and ice melt of Thwaites Glacier, West Antarctica. *Sci. Adv.* **5**, (2019).

46. Smith, J. A. *et al.* Sub-ice-shelf sediments record history of twentieth-century retreat of Pine Island Glacier. *Nature* **541**, 77–80 (2017).

47. Tinto, K. J. & Bell, R. E. Progressive unpinning of Thwaites Glacier from newly identified offshore ridge: Constraints from aerogravity. *Geophys. Res. Lett.* **38**, (2011).

48. Jourdain, N. C. *et al.* Simulating or prescribing the influence of tides on the Amundsen Sea ice shelves. *Ocean Model.* **133**, 44–55 (2019).

49. Holland, D. M. & Jenkins, A. Modeling Thermodynamic Ice–Ocean Interactions at the Base of an Ice Shelf. *J. Phys. Oceanogr.* **29**, 1787–1800 (1999).

50. Padman, L., Fricker, H. A., Coleman, R., Howard, S. & Erofeeva, L. A new tide model for the Antarctic ice shelves and seas. *Ann. Glaciol.* **34**, 247–254 (2002).

51. Robertson, R. Tidally induced increases in melting of Amundsen Sea ice shelves. **118**, 3138–3145 (2013).

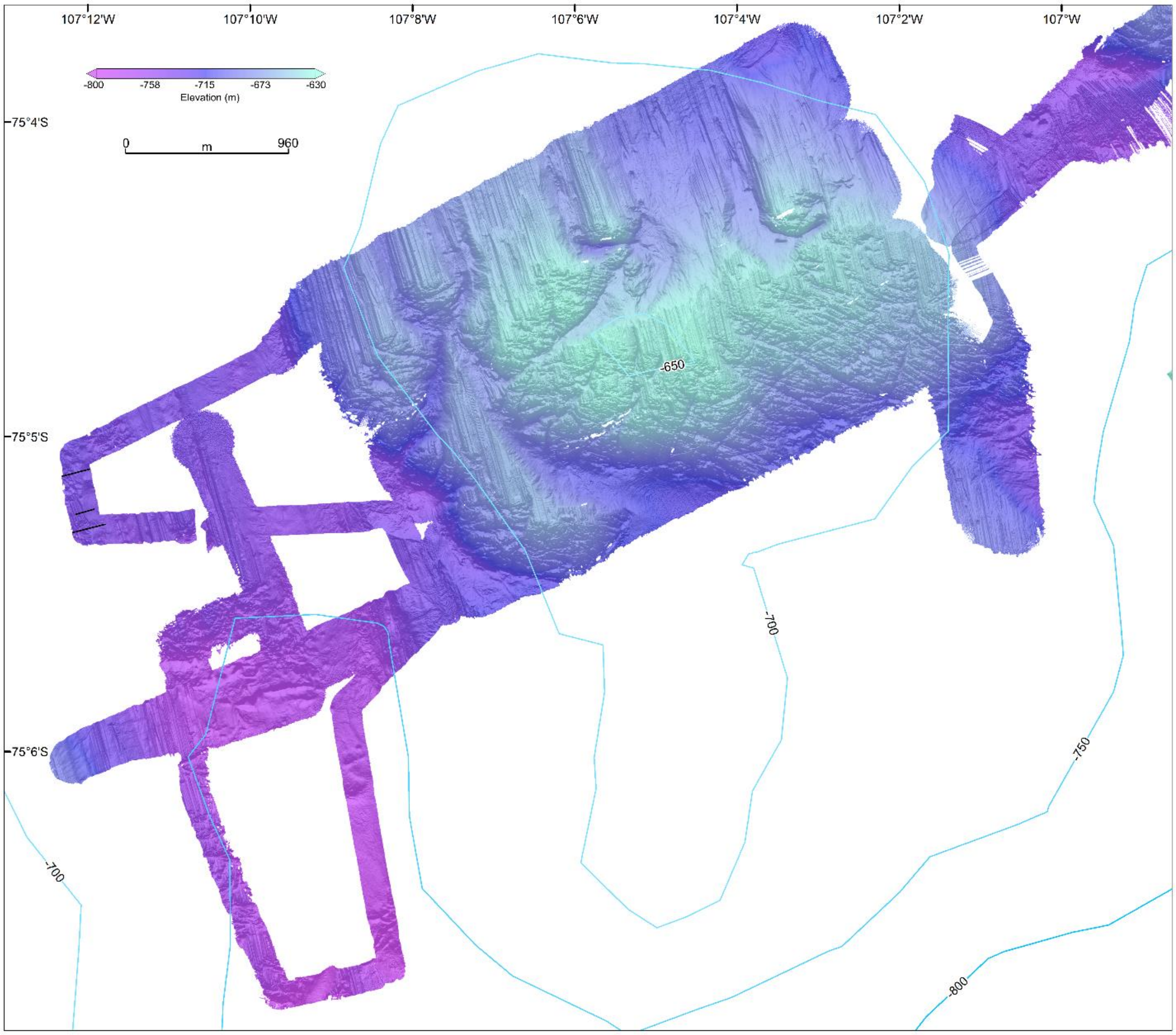

Fig. S1. Full resolution multibeam bathymetry data for AUV mission 009 on the 'bump'. Extended imagery as a supplement to Figure 1D of main manuscript. Swath bathymetry data acquired using a Kongsberg EM2040 AUV-integrated multibeam echo sounder. 1.5 m grid resolution.

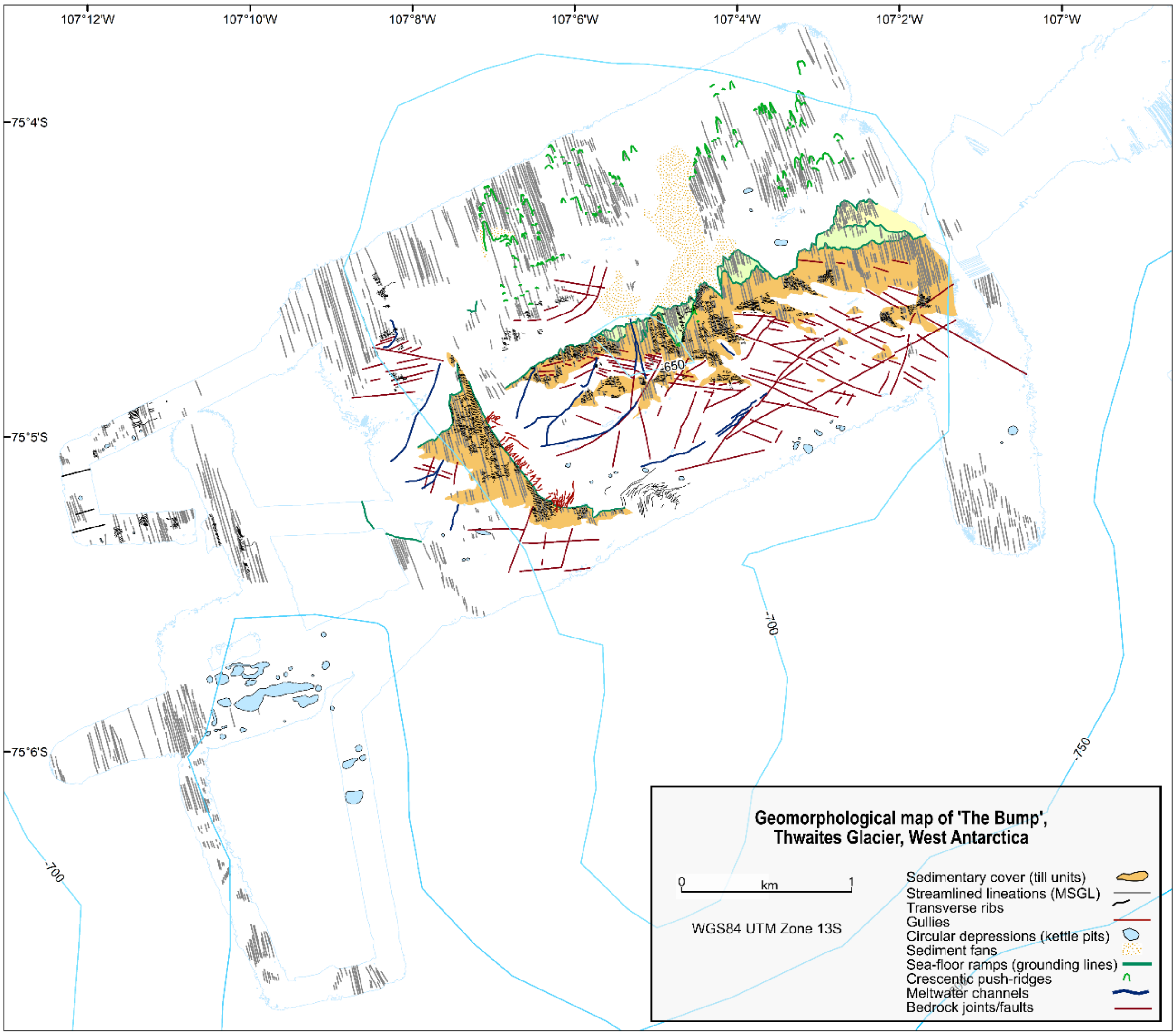

Fig. S2. Large-scale geomorphological map of 'the bump' and surrounding region. Extended mapping as supplement to Figure 2A of main manuscript.

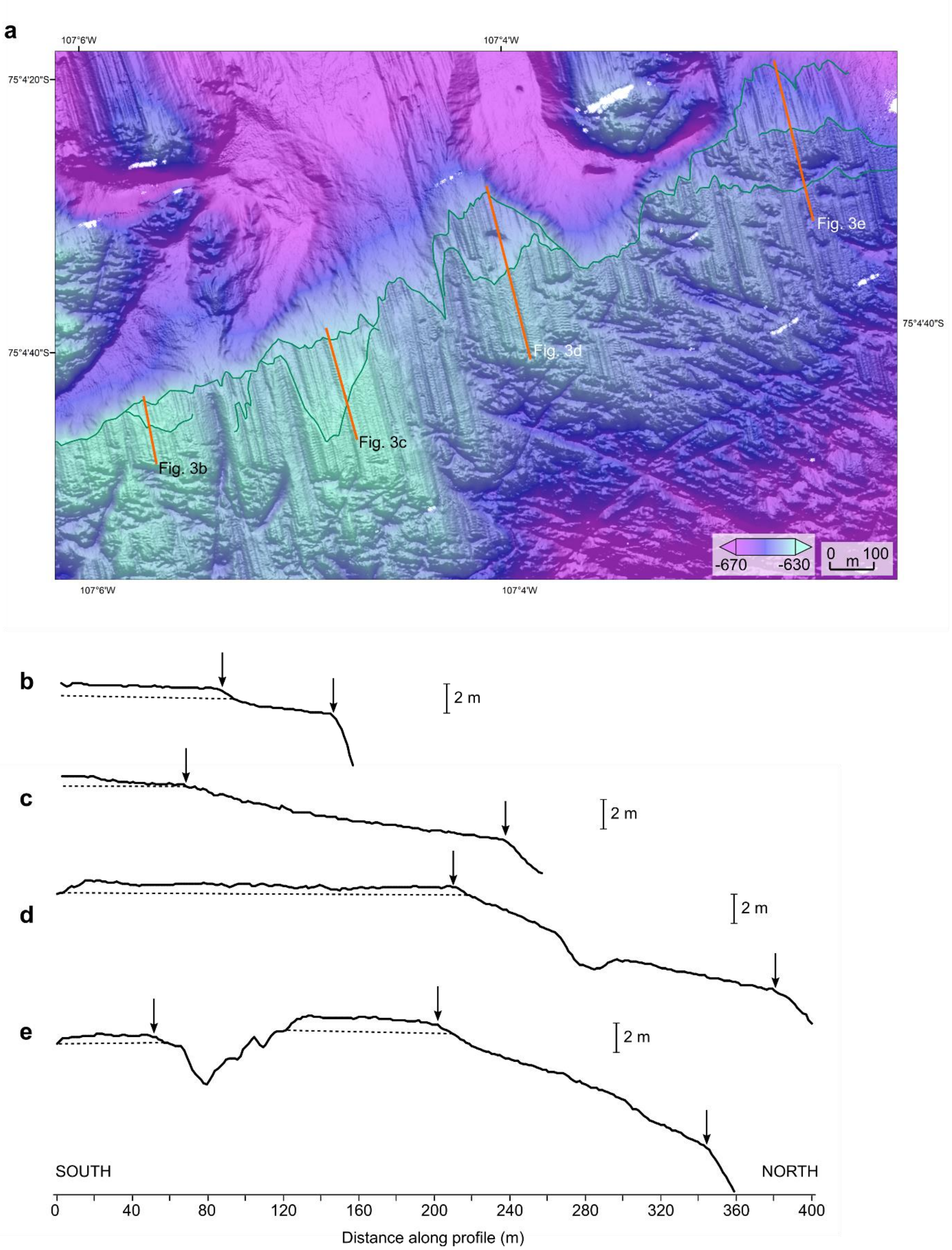

Fig. S3. **Topographic profiles through the grounding zone surfaces and grounding-line limits (arrowed) on the 'bump'.**

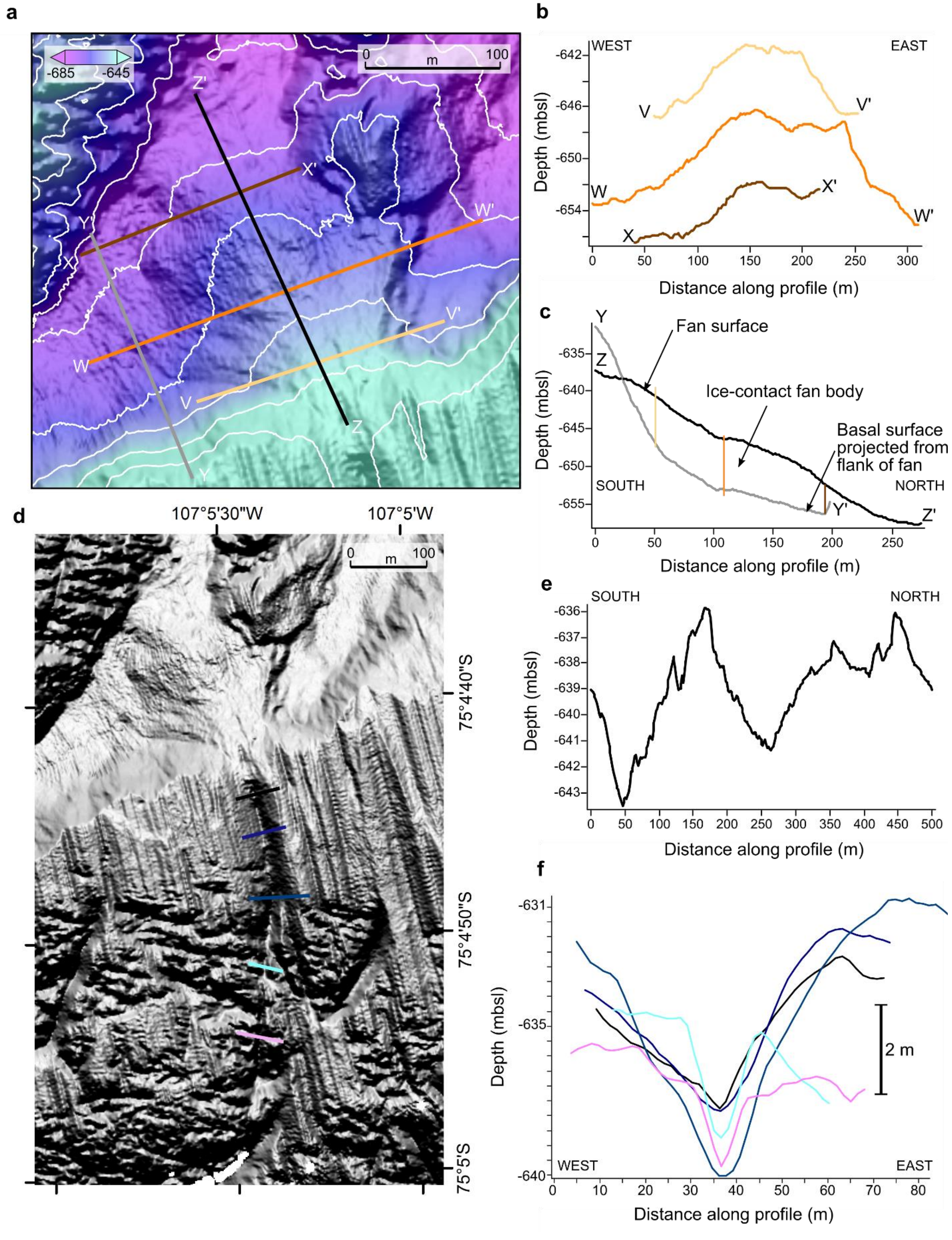
a
-685
-645
0
m
100
Z'
X'
W'
Y'
X
V'
W
V
Z
Y
b
WEST
EAST
V
V'
W
W'
X
X'
Depth (mbsl)
Distance along profile (m)
c
Y
Z
Fan surface
Ice-contact fan body
Basal surface projected from flank of fan
SOUTH
NORTH
Y'
Z'
Depth (mbsl)
Distance along profile (m)
d
107°5'30"W
107°5'W
75°4'40"S
75°4'50"S
75°5'S
e
SOUTH
NORTH
Depth (mbsl)
Distance along profile (m)
f
WEST
EAST
2 m
Depth (mbsl)
Distance along profile (m)

Fig. S4 (overleaf). **Multibeam swath bathymetry data showing details of a connected subglacial meltwater conduit and ice-contact fan on the northern margin of the bump.** (A) Detailed multibeam swath bathymetry image of the ice-proximal fan with 5-m contours illustrating the characteristic bulge in the northern slope of the bump. (B) Cross-section profiles through the upper, mid, and lower portion of the fan body. (C) Representative cross-section of the fan surface and base, showing the overall internal form of the deposit. (D) Hillshade derivation of the multibeam bathymetry illustrating the linked channel-fan system. (E) Long-axis (thalweg) of the channel, demonstrating an undulating, rugged, and generally seaward-shoaling morphology. (F) Cross-section profiles at stages along the channel length depicting a narrow and deeply-incised form.

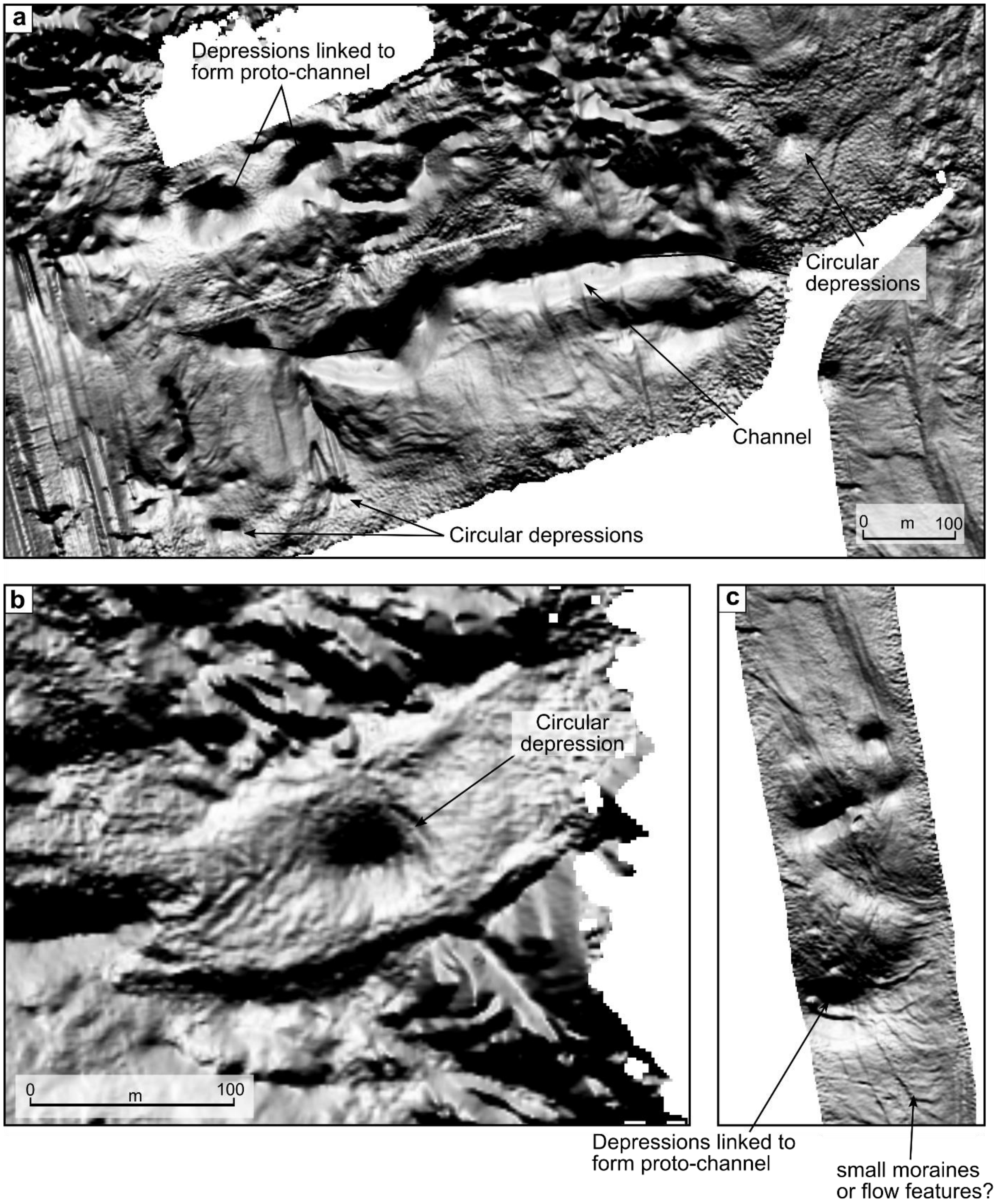

Fig. S5. Greyscale hillshade models of high-resolution multibeam bathymetry illustrating various examples of circular depressions in the study region. Some of the depressions occur in bands or chains and appear linked to form proto-channels (A). Other depressions are isolated (A and B). Extremely fine-scale moraines or flow features appear as spiralling ridges at the flanks of several depressions (B and C). All features of this type are found at water depths of 700-800 m.

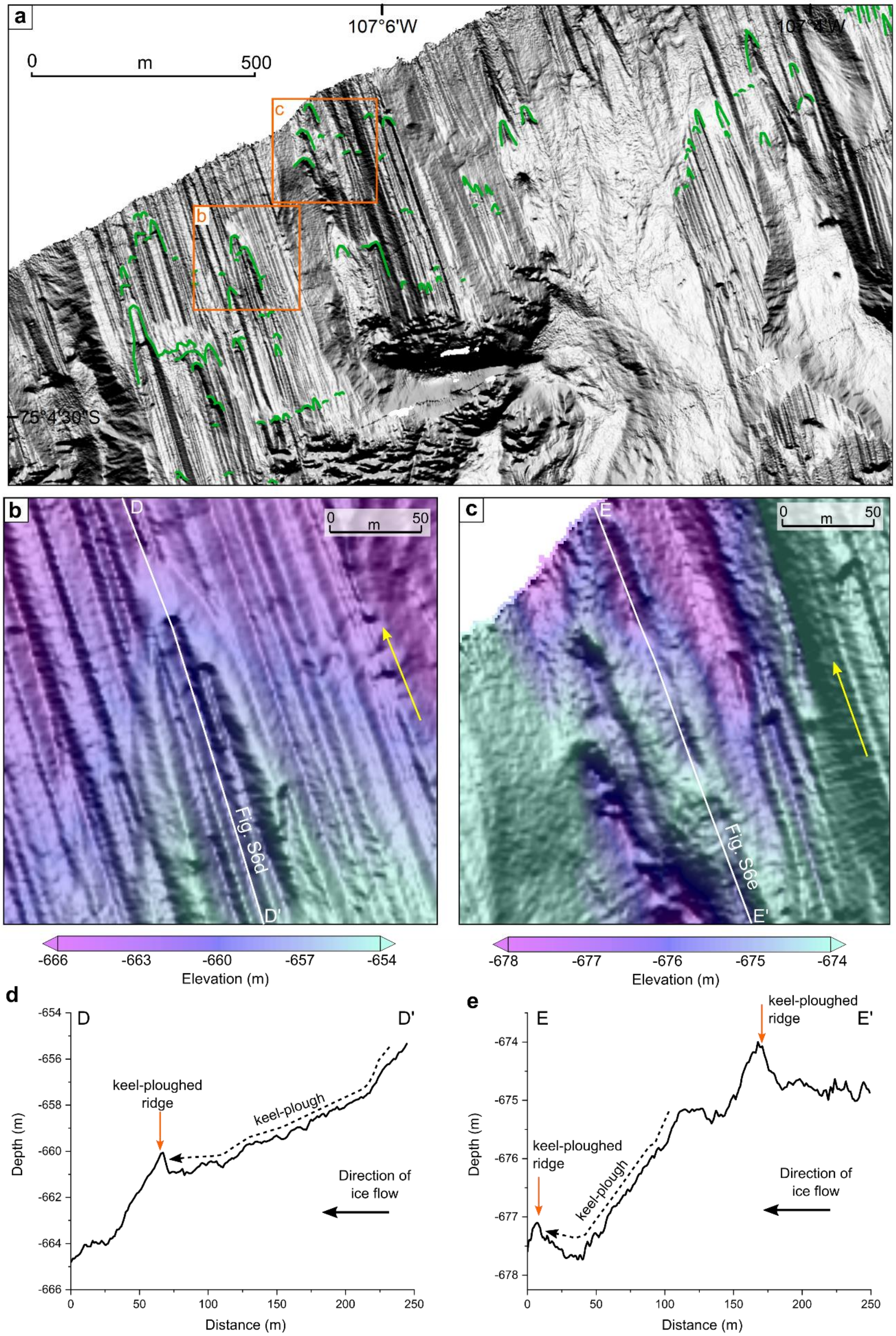
a
107°6'W
107°4'W
0
m
500
c
b
75°4'30"S
b
D
0
m
50
Fig. S6d
D'
-666
-663
-660
-657
-654
Elevation (m)
c
E
0
m
50
Fig. S6e
E'
-678
-677
-676
-675
-674
Elevation (m)
d
D
D'
keel-ploughed ridge
keel-plough
Direction of ice flow
Depth (m)
Distance (m)
e
E
E'
keel-ploughed ridge
keel-ploughed ridge
keel-plough
Direction of ice flow
Depth (m)
Distance (m)

Fig. S6 (overleaf). Multibeam swath bathymetry data examples of geomorphological features interpreted as keel-formed grounding-line moraines, mapped extensively in deeper water north of the bump. (A) Greyscale hillshade model of high-resolution multibeam bathymetry for the northern part of the survey area showing mapped push-ridges (green) on the distal slopes (tails) of bedrock crags. (B-E) Detailed examples and long-section bathymetric profiles showing the three-dimensional ploughed ridge geometries.

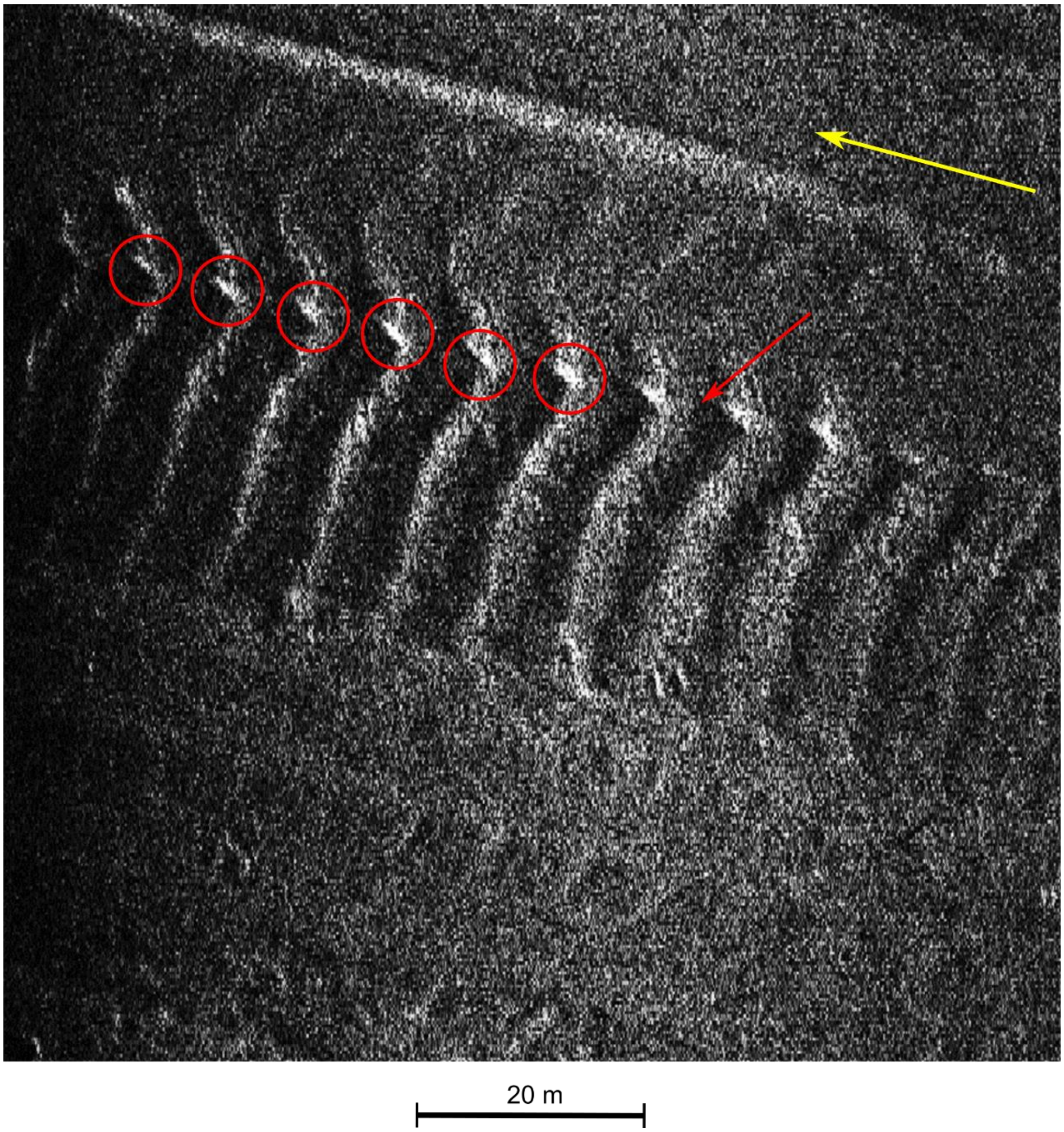

Fig. S7. High-frequency sidescan data acquired by the Hugin AUV on top of the bump. Figure shows details of ‘ribs’ overprinting streamlined subglacial lineations (yellow arrow shows flow direction). The ‘beading’ of lineation longitudinal ridges is clearly visible (red circles), as is the obliteration of the lineation in the zones intervening the ribs (red arrow). Both observations support the superimposition of lineations by the ribs, and a process of deformation and extrusion of existing substrate to form the rib features. Ribs are also continuous across more than one ridge-groove.

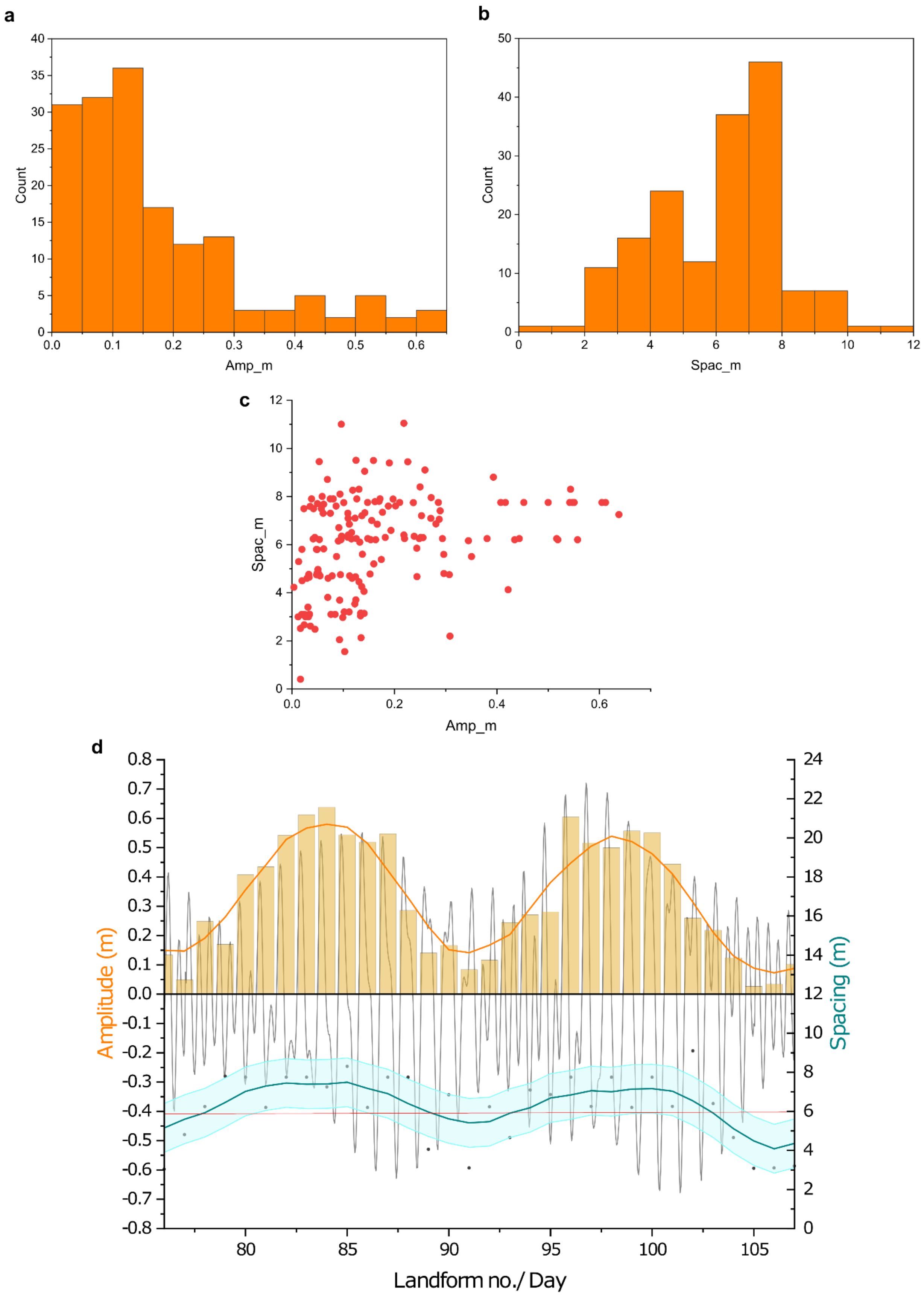
a
Count
Amp_m
b
Count
Spac_m
c
Spac_m
Amp_m
d
Amplitude (m)
Spacing (m)
Landform no./ Day

Fig. S8 (overleaf). Geometric data for the longest series of ribs in the dataset. Histograms of rib amplitude (A) and spacing (B). (C) Plot of amplitude vs spacing for the rib series illustrating the strong co-variance between the two metrics. (D) Tidal model predictions for Thwaites Glacier region (grey) covering two full spring-neap tidal cycles overlain with landform amplitude (m; orange bars) and landform spacing (blue dots) data formed in series and scaled to assume that 1 landform = 1 tidal day. Blue and orange lines are 7-point weighted averages of the landform data, and light blue shaded area is 1 standard deviation. Note that diurnal cycles in the tide model appear dominant especially in the lead in, during, and waning of the spring tides. Semi-diurnal constituents appear in a more complex tidal signal around neap tides. This complexity may explain some of the small-scale potential disparity between the landform series and the tide model because there is a chance of more than one rib forming per day during the neap tide.

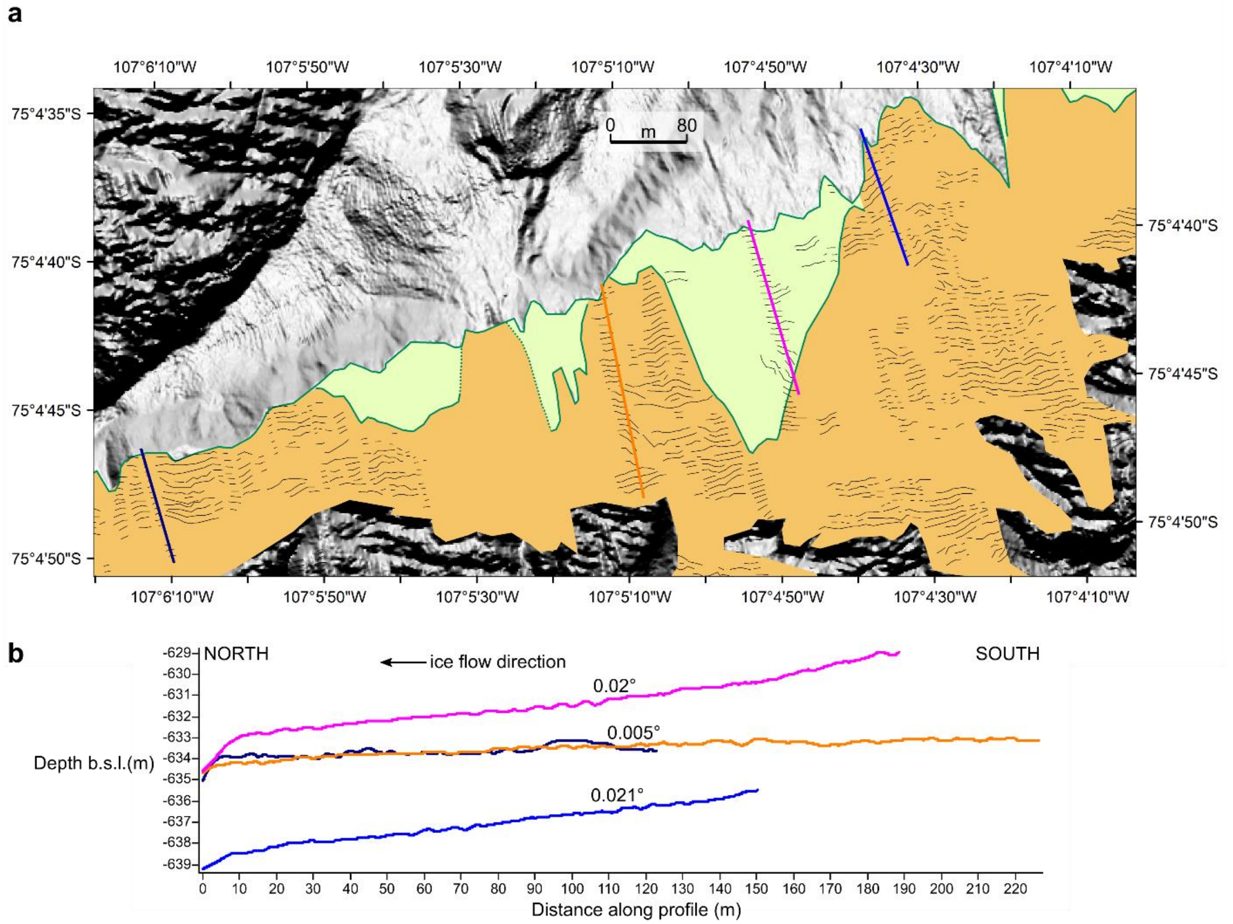

Fig. S9. **Topographic elevation profiles demonstrating the shallow seaward dip of surfaces on which many ribs are mapped.** (A) Multibeam bathymetry hillshade overlain with mapped grounding zone surfaces. The orange shaded polygon demarcates the most recent region of ice-sheet grounding and grounding-line retreat (referred to in the paper as GZ3). Black lines are ribs mapped from geophysical datasets. (B) Four sea-floor topographic profiles showing the very low-gradient, seaward dip of the ocean floor on which the ribs were formed.

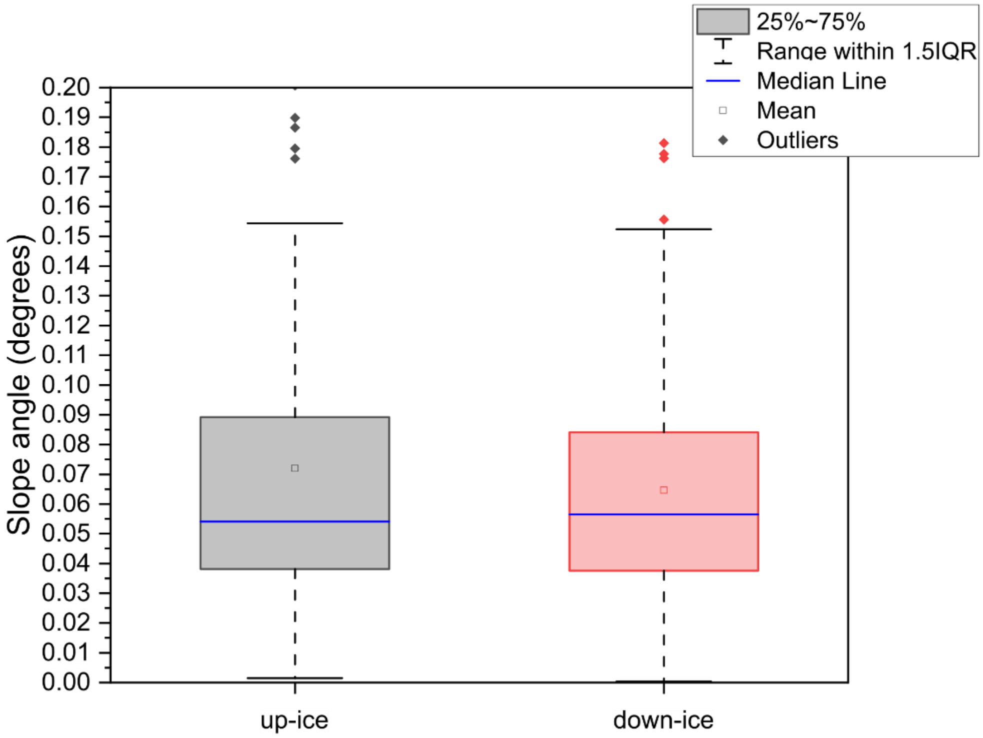

Fig. S10. Box plots illustrating slope analysis for up-ice and down-ice flanks of ribs. Figure demonstrates the broadly symmetric nature of the landforms across a large dataset. Data represent the northern 1 km of the profile shown in Figure 4a of the main manuscript. Slopes are derived from analysis of the peak to trough height and distance assuming each rib is straight sided and after regional relief has been removed from the dataset. In reality, locally, ribs may show higher and slightly more variable slopes. For reference, a 17 cm high rib with a 3 m peak-to-trough spacing (1/2 λ) has a slope corresponding to the median (c. 0.055°); a 70 cm high rib with a 3.5 m peak-to-trough spacing has a slope corresponding to the observed maximum slope (c. 0.19°).

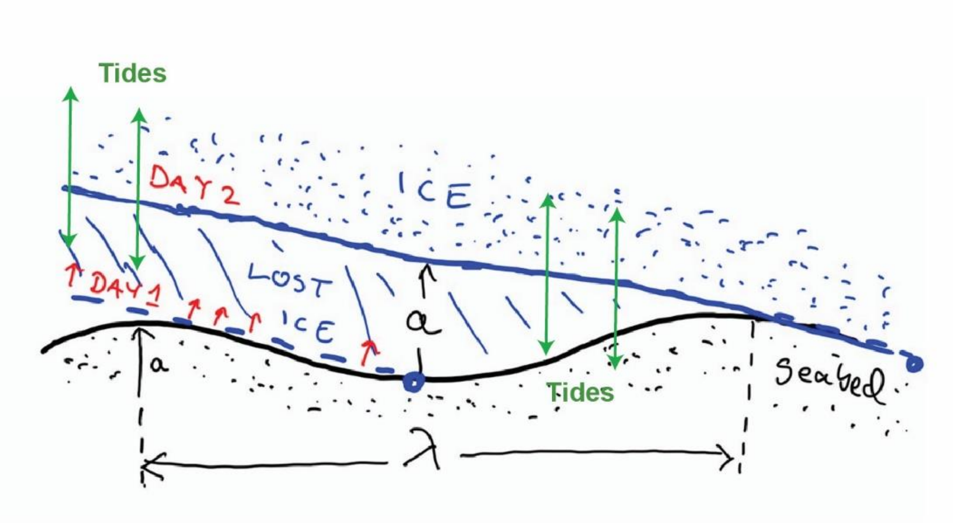

Fig. S11: Sketch of the grounding zone geometry and minimum basal melt. Red arrows indicate basal melt, green arrows indicate vertical movement due to tides. The distance between high-tide marks is λ and *a* is the height of the corrugations. Graphic does not depict the opening and closing of the grounding zone cavity landward as the tide rises and falls.

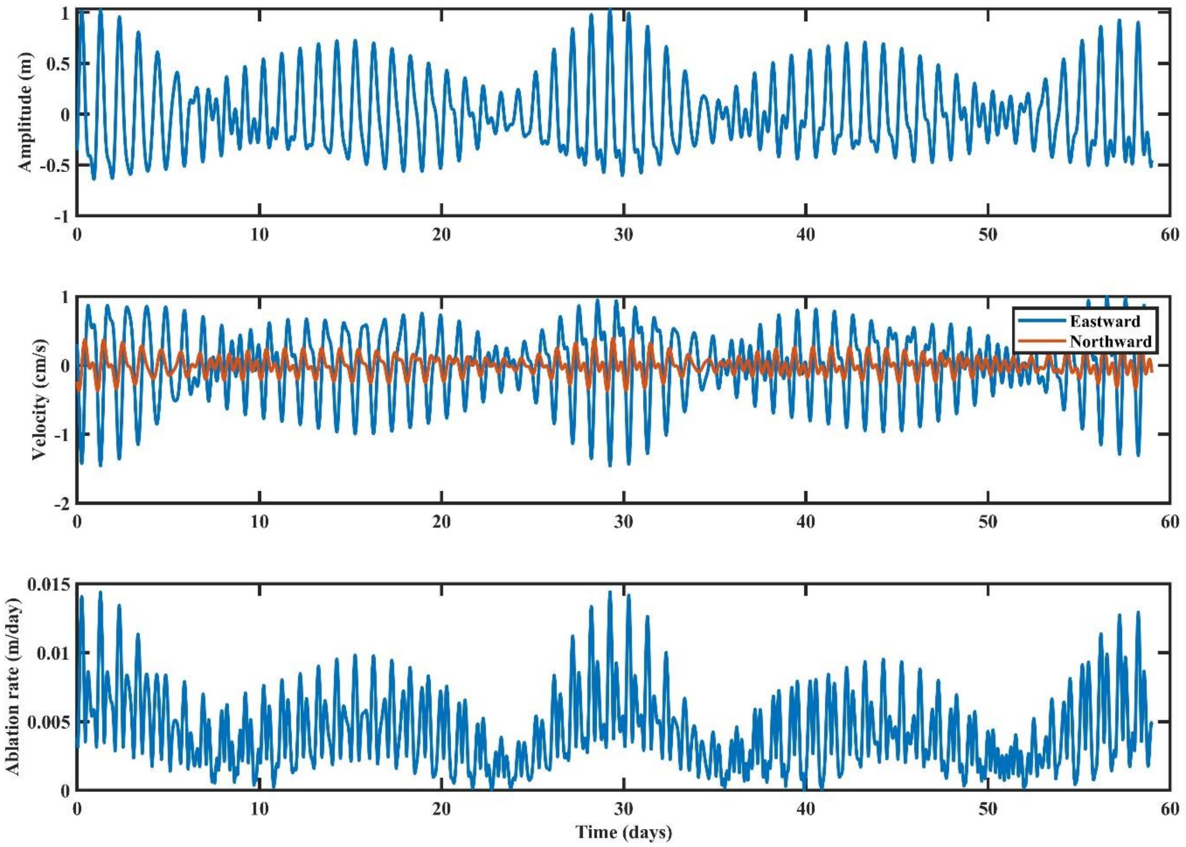

Fig. S12. Results of the CATS2008 tide model[50]. (top) Amplitude (center) Flow velocity (bottom) Ablation rate calculated using (5) and (6).

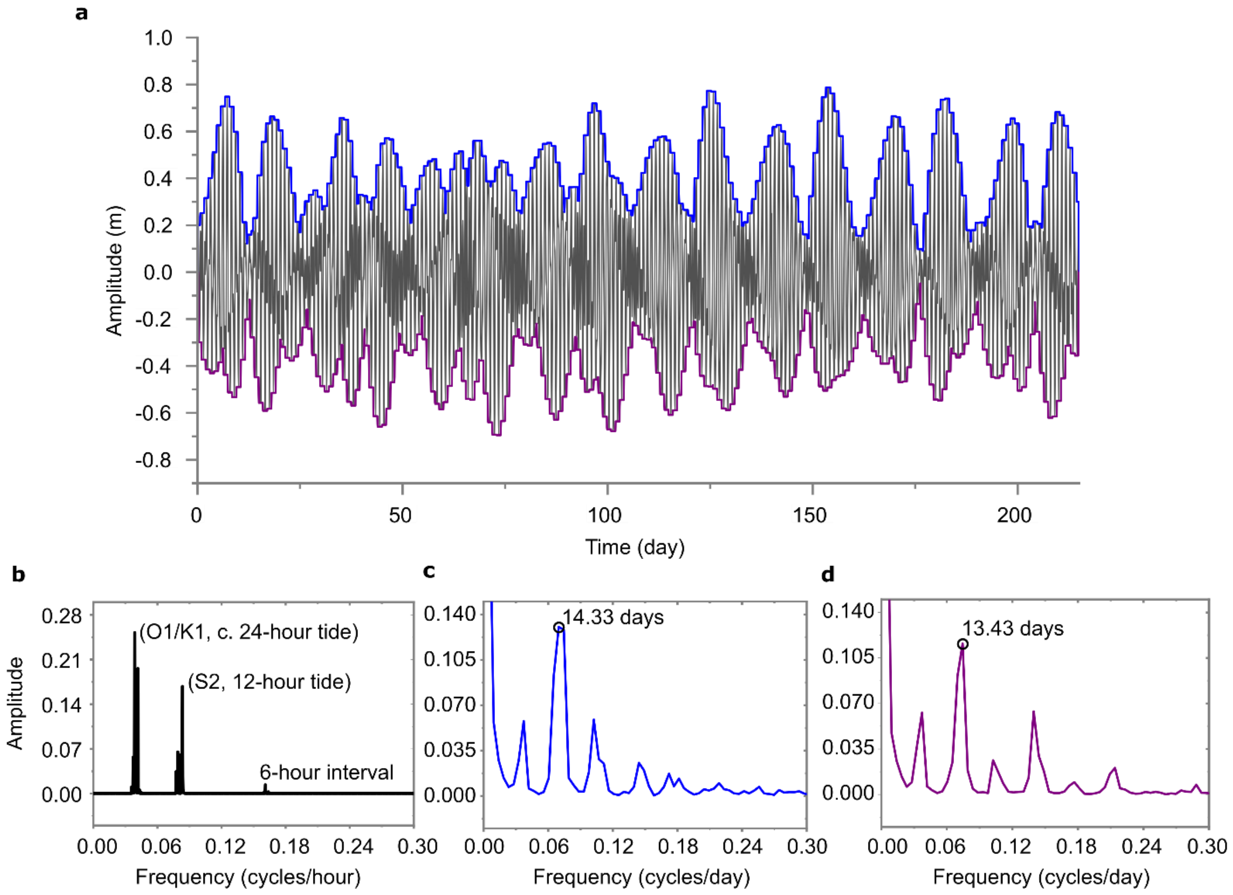

Fig. S13. Tidal model analysis at Thwaites Glacier. A) Tidal data for the ocean proximal to Thwaites Glacier for a period covering 215 days of 2020 (grey; hourly data shown). 99th (blue) and 1st (purple) percentile filtered data with a 24-point window are overlaid, tracking the daily high/low tidal signal through the dataset, respectively. B-D) Shows the corresponding FFT periodograms for the three datasets in 'a', with peak frequencies labelled (colors correspond to those in 'a').
